# Hyperbranched Dynamic Crosslinking Networks Enable Degradable, Reconfigurable, and Multifunctional Epoxy Vitrimer

**DOI:** 10.1002/advs.202306350

**Published:** 2023-11-07

**Authors:** Yuanbo Zhang, Hongxia Yan, Ruizhi Yu, Junshan Yuan, Kaiming Yang, Rui Liu, Yanyun He, Weixu Feng, Wei Tian

**Affiliations:** ^1^ Shaanxi Key Laboratory of Macromolecular Science and Technology Xi'an Key Laboratory of Hybrid Luminescent Materials and Photonic Device School of Chemistry and Chemical Engineering Northwestern Polytechnical University Xi'an 710129 China

**Keywords:** degradation and cycling of thermosets, dynamic crosslinking networks, hyperbranched polymers, vitrimers

## Abstract

Degradation and reprocessing of thermoset polymers have long been intractable challenges to meet a sustainable future. Star strategies via dynamic cross‐linking hydrogen bonds and/or covalent bonds can afford reprocessable thermosets, but often at the cost of properties or even their functions. Herein, a simple strategy coined as hyperbranched dynamic crosslinking networks (HDCNs) toward in‐practice engineering a petroleum‐based epoxy thermoset into degradable, reconfigurable, and multifunctional vitrimer is provided. The special characteristics of HDCNs involve spatially topological crosslinks for solvent adaption and multi‐dynamic linkages for reversible behaviors. The resulting vitrimer displays mild room‐temperature degradation to dimethylacetamide and can realize the cycling of carbon fiber and epoxy powder from composite. Besides, they have supra toughness and high flexural modulus, high transparency as well as fire‐retardancy surpassing their original thermoset. Notably, it is noted in a chance‐following that ethanol molecule can induce the reconstruction of vitrimer network by ester‐exchange, converting a stiff vitrimer into elastomeric feature, and such material records an ultrahigh modulus (5.45 GPa) at −150 °C for their ultralow‐temperature condition uses. This is shaping up to be a potentially sustainable advanced material to address the post‐consumer thermoset waste, and also provide a newly crosslinked mode for the designs of high‐performance polymer.

## Graphical abstract

Hyperbranched dynamic crosslinking networks (HDCNs) convert a thermoset network from permanently three‐dimensional‐structure to dynamically topological‐crosslinked architecture, programming a petroleum‐based thermoset into degradable, reconfigurable and multifunctional vitrimer. They are capable of room‐temperature degradation to solvent, and exhibit reconfigurability from stiff vitrimer to elastomer. Vitrimers also display enhanced modulus, toughness, flame‐retardancy, and high transparency when compared to native commodity thermosets.

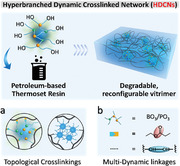

## 1 Introduction

Plastic products have become deeply rooted in human society. Globally, more than 100 million tons of plastic waste are annually discarded into the environment, which are landfilled, incinerated, or discharged into the ocean, resulting in serious ecological damages.^[^ ^1^ ^,2]^ Epoxy resins (EPs), derived from petroleum, are cost‐effective thermosetting materials that are ubiquitously used in aerospace, transportation, architecture, and electronic and electrical devices.^[^ ^3^, ^4^ ^]^ However, thermosets have a limited capacity to degrade and recycle than thermoplastics or supramolecular plastics due to their three‐dimensional (3D) crosslinked structure.^[^ ^5^, ^6^, ^7^, ^8^ ^]^ Once cured, it will cause permanent plastic wastes that require hundreds of years to naturally clean up. To resolve the problem, tremendous efforts are still necessitated toward degradable and closed‐loop cycling epoxies as alternatives to traditional ones,^[^ ^9^, ^10^ ^]^ such as developing bio‐based monomers.^[^ ^11^ ^]^ However, there still remain intractable challenges for these structures such as their high cost, and how to make a trade‐off between high structural strength and easy degradability, as well as further realize multifunctional material purposes.^[^ ^12^, ^13^ ^]^

Alternatively, dynamic‐covalent and/or non‐covalent crosslinking strategies are particularly attractive for the designs of next‐generation materials with healable and chemical recycling properties.^[^ ^14^, ^15^, ^16^, ^17^, ^18^, ^19^, ^20^, ^21^, ^22^ ^]^ This fact derives a new class of polymers named vitrimers, which merge the combinative features of thermoplastics and thermosets.^[^ ^23^, ^24^, ^25^, ^26^, ^27^, ^28^ ^]^ Epoxy vitrimers are emerging and explored as the time demand for programming degradable and recyclable epoxies and even their thermosets.^[^ ^29^, ^30^ ^]^ Vitrimers are generally constructed by dynamic‐covalent bonds linked with polymer networks (DCPNs), wherein bond reversion is driven by catalysts or external stimuli.^[^ ^31^, ^32^, ^33^ ^]^ In many cases, the control exerted over the crosslinked structure by governing dynamic covalent bonds tends to compromise their natural thermosetting virtues (for example, thermal and chemical resistance, and mechanical robustness). Therefore, a holistic perspective involving supramolecular modes and covalent adaptable modes will contribute to an in‐depth understanding of crosslinking behavior from chemical bonds to linear monomers, and even to the whole crosslinked structure of polymer networks.^[^ ^34^, ^35^, ^36^, ^37^ ^]^

Unlike classical linear polymers, hyperbranched polymers (HBPs) possess spatial molecular configurations with 3D‐branched architecture and abundant external terminals.^[^ ^38^, ^39^ ^]^ They can be easily functionalized for polymer modification and are a well‐established strategy for achieving high strength and toughness of thermoset polymers.^[^ ^40^ ^]^ Considerable progress has been made in developing various hyperbranched structures and prototyping them in relevant functional applications.^[^ ^41^, ^42^, ^43^, ^44^, ^45^, ^46^ ^]^ Benefiting the virtue of their hyperbranched architecture, HBPs with broad intramolecular cavities can reduce network density to accommodate solvent molecules, thus driving dynamic behaviors. Hence, they hold promise for tailoring degradable and in‐processable vitrimers from native thermosets. The interpenetrating networks formed by HBPs and epoxy matrices, characterized by network topologies and dynamic linkages, are poised to bestow upon thermoset polymers unique and exceptional characteristics. Moreover, they offer ease of functionalization, particularly in compensating for the lack of mechanical robustness.

**Figure 1:**
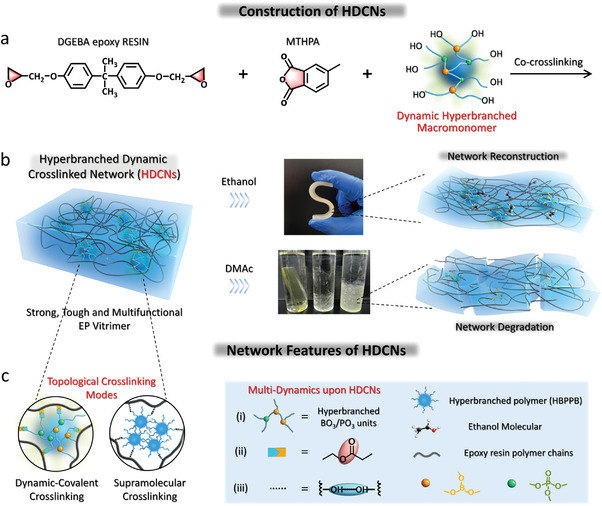
The conceptual basis of this work. a) The HDCNs forming strategy via a hyperbranched macromonomer bearing ─OH terminal and dynamic bonds to co‐crosslink with industrial thermoset epoxy. b) The schematic model describing HDCNs enables a strong and tough epoxy vitrimer with reconfigurable and degradable performance. c) Network features: from double topologically crosslinking modes to reduce network density for adapting solvents, as well as multiple dynamic linkages involving covalent PO_3_/BO_3_, ester bonds, and non‐covalent hydrogen bonds.

Herein, we report hyperbranched dynamic crosslinking networks (HDCNs) to program an industrial thermoset into a degradable and reconfigurable vitrimer. The conceptual basis underlying this approach is depicted in **Figure** **1**. The HDCNs are structured with multiple dynamics via introducing a hyperbranched macromonomer (HBPPB) bearing dynamic units and reactive terminals. Through the integration of boron and phosphorus units, the molecular configuration can be well stretched into a topology, exploiting the planar‐like nature (sp2 hybridization) of boron and the sp3 hybridization of phosphorus esters. This hybridized structure establishes covalent crosslinks with DGEBA monomer (ring‐opening) and anhydride monomer (forming ester bond), as well as supramolecular crosslinking via H‐bonding (Figure 1c). Consequently, the installation of HDCNs makes a thermoset network from a permanently 3D structure to a dynamic topologically crosslinked architecture, wherein the branching architecture benefits an expanded crosslinking network (low‐crosslink‐density, Figure 4c) that is capable of adapting solvent molecule entrance to drive the dynamic bond exchange. Such structural features render HDCNs susceptible to mild solvent‐dissolved degradation (room temperature, Figure 1b) and confer transformative material property^[^ ^47^, ^48^, ^49^ ^]^ from a rigid vitrimer to an elastomer upon heating in ethanol (See Movie S1, Supporting Information). More attractively, the after‐treated vitrimer showcases a suprahigh modulus (5.45 GPa) at extremely low temperature (−150 °C), posing them and even their composites supranormal uses for astrospace, superconducting energy storage, medical equipment, and beyond. Furthermore, the vitrimer exhibits high flexural modulus and toughness, and excellent thermal stability, among other high added values, demonstrating an extension of existing vitrimers and vitrimer properties. In light, this design principle via HDCNs may hold promise for the breakthrough of conventional concepts toward the next generation of sustainable and advanced thermoset polymers.

## 2 Results and Discussion

### 2.1 Synthesis and Characterization of HDCNs

The synthesis of HDCNs is straightforward via introducing a hyperbranched dynamic macromonomer into a traditional epoxy thermoset network as a dynamic crosslinker. The hyperbranched macromonomer (HBPPB) was synthesized via A_2_+B_2_+C_3_ polycondensation (Section S1.3a, Supporting Information). In parallel, a linear poly‐phosphate/borate structure (LPPB), a hyperbranched polyborate (HBPB), and a hyperbranched polyphosphate (HBPP) were synthesized as controls to compare with HDCNs. Synthesis details are described in Sections S1.4 and S1.5 (Supporting Information). The molecular structure of the synthetic polymers was characterized using multiple analytical techniques, including Fourier transform infrared (FT‐IR), ^1^H, ^13^C, and ^31^P nuclear magnetic resonance (NMR) spectroscopies, gel permeation chromatography (GPC) (see Section S1.3b, Supporting Information). The concentration of hydroxyl groups (─OH) in HBPPB was quantified via a standard titration experiment, yielding a value of 7.97 × 10^−3^ mol g^−1^ (Section S1.3c and Table S2, Supporting Information), indicating the high abundance of ─OH groups.

The polymer matrix comprised of a petroleum‐based epoxy (diglycidyl ether bisphenol A, DGEBA, E51) a commercially used anhydride‐type curing agent (methyl tetrahydrophthalic anhydride, MTHPA), and a certain amount of HBPPB. A facile thermosetting workflow was employed to cast EP‐x samples, where *x* represents the mass concentration of HBPPB. The curing performance was evaluated using isothermal differential scanning calorimetry (Figure S21c, Supporting Information), which showed a homogeneous and single peak among all pre‐polymer systems, indicating well compatibility among the three constituents. The natural EP and EP‐9 underwent full curing as proved in its time‐sweep curves by rheology (Figure S22, Supporting Information). For subsequent analysis, the epoxy samples containing HBPPB equal to or greater than 9 wt.% were donated as vitrimers due to their degradable and/or reconfigurable dynamic behavior. The other samples were categorized as thermosets due to their predominant thermoset nature. To facilitate comparative investigations, epoxy vitrimers containing varying mass fractions of LPPB, HBPB, and HBPP were prepared using the same procedure as HDCNs, and a comparison of their material properties is presented in Sections S2.5 and S2.6 (Supporting Information).

The dynamic nature of HDCNs was characterized extensively through stress relaxation (see Section S2.3, Supporting Information) and creep‐recovery test (Section S2.4, Supporting Information) in tensile mode.^[^ ^50^, ^51^, ^52^, ^53^, ^54^ ^]^ In EP‐12, the time‐ and temperature‐dependent modulus revealed a partial relaxation to a plateau regime that deviates from classical linear‐viscoelastic vitrimers. This unique relaxation signifies that HDCNs not only behave with liquid‐like viscoelasticity due to the activation of dynamic exchange above the topology‐freezing transition temperature (*T*_v_, described by Arrhenius and Williams−Landel−Ferry law), but also retain the resilience and recovery capabilities like an elastic body subjected to Hooke's law. By fitting the results on an Arrhenius plot, a linear correlation between ln(τ*) (relaxation time) and 1000/T (inverse temperature) was observed, enabling the calculation of an activation energy (*E*_a_) of 78.1 kJ mol^−1^ and *T*_v_ of 71.7 °C (Figures S24 and S25, Supporting Information). It is noteworthy that several examples of vitrimers with *T*_g_ > *T*_v_ have been reported.^[^ ^55^, ^56^ ^]^ Consequently, the material exhibits dynamic vitrimer behavior, albeit with reduced dynamic capabilities compared to fully relaxed vitrimers. Nonetheless, it demonstrates exceptional dimensional stability and mechanical strength even above the glass‐transition temperature (*T*_g_) and *T*_v_. This behavior can be attributed to the presence of permanently crosslinked sites and potentially relates to the network topologies, thereby underlying further understanding of vitrimers and their dynamic behaviors.

### 2.2 Cleavable and Topological Crosslinks Control over Degradable HDCNs

**Figure 2:**
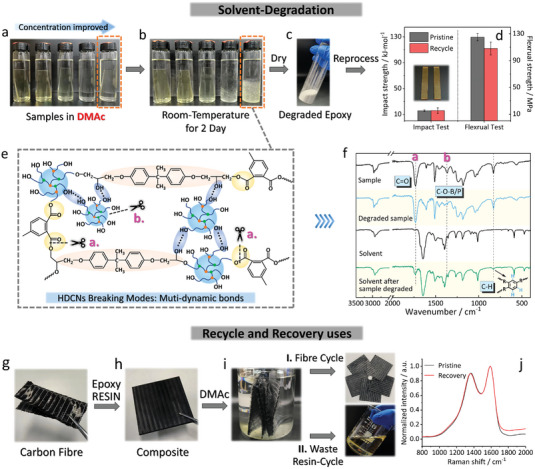
Degradation and cycling case of HDCNs. a,b) Samples immersed in DMAc for the solvent‐degradation varying the concentration of HBPPB. c) degradation product underwent drying and grinding into epoxy powder. d) cycling epoxy powder with virgin epoxy resin at a ratio of ≈1:5 for comparable mechanical strength. e) HDCNs braking modes: multiple cleavable units upon the HDCNs. f) IR spectrum before‐ and after‐ degradation. g–i) cycling case for end‐of‐use carbon fiber and epoxy resin from composite. j) Raman spectra of the recovered carbon fiber.

Thermoset polymers are generally considered non‐degradable and hard to reprocess, particularly when in comparison to thermoplastic or weakly crosslinked supramolecular plastics.^[^ ^57^, ^58^ ^]^ However, inserting topological crosslinks and cleavable bonds into the thermoset network via HDCNs leads to the formation of vitrimers that exhibit distinctive behavior upon exposure to solvents (**Figure** **2**; Section S1.7, Supporting Information). In this study, a commonly used aprotic solvent (dimethylacetamide, DMAc) was employed to fully immerse the samples. EP‐12, as the most prominent case for degradation, underwent swelling and fragmentation within a mere 2 days at room temperature (Figure 2a,b), in stark contrast to the natural thermoset EP, which retained its intact and rigid shape. Moreover, an increasing concentration of HBPPB can accelerate the process. In fact, a rapid degradation was observed within 6 hours when the system temperature was raised to 95 °C in DMAc (Figure S13, Supporting Information). Based on these differences, we infer that the solvent‐assisted degradation was associated with cleavable crosslinks in HDCNs.

Unlike classic thermoset networks or degradable covalent networks, most of them are specialized for the degradation of weakly crosslinked materials such as thermoplastics and supramolecular plastics but are often helpless for highly crosslinked thermoset networks.^[^ ^59^, ^60^ ^]^ Whereas HDCNs benefit the stretching of molecular configurations for topological crosslinking purposes, which not only consists of several cleavable units but, more importantly, enables a topologically expanded network interspace that allows solvent molecular entrance to drive dynamic behaviors. The crosslinking density of HDCNs is relatively lower than their thermoset EP counterpart (Figure 4c). To assert the breakages when degrading, a schematic model describes the possible mechanisms in Figure 2e. Such a structure model encompasses three types of cleavable bonds. The first involves two kinds of carbonic ester bonds (mark a) that result from the reaction between the anhydride with HBPPB and the epoxy monomer (Figure S10, Supporting Information). The second breakage is attributed to the boronic ester group (BO_3_)^[^ ^53^, ^61^, ^62^ ^]^ and the phosphate group (PO_3_)^[^ ^63^ ^]^ upon the molecular backbone of the hyperbranched crosslinker. Third, non‐covalent H‐bonds are also susceptible to cleavage when the solvent is diffused.

To monitor the aforementioned breakages, both EP‐12 and the solvent were subjected to FT‐IR analysis, as shown in Figure 2f. The results reveal distinct spectral features, notably the emergence of a newly detected C═O absorption (mark a) and C─H signals within the substituted benzene moiety in the DMAc spectrum after degradation, indicating the species of anhydride‐contained substrates are partially dissolved in DMAc, primarily indicating the cleavage of these carboxyl ester bonds. Furthermore, the degraded sample exhibits a diminished C─O─B/P absorption (mark b), which corresponds to the depolymerization of HBPPB. Complementary insights from the X‐ray photoelectron spectroscopy (XPS, details in Section S2.8, Supporting Information) reveal that the beta‐hydroxy ester (formed through the reaction of the anhydride with ‐OH and epoxy groups) underwent breakage, as evidenced by its heightened and broader O─H/COOH intensity in the O1s spectra, as well as the relatively lower signals of C═O and C─O─C in the C1s spectra. More importantly, the degraded sample shows very weak signals of B and P in its high‐resolution XPS, thus further confirming the depolymerized HBPPB. Consequently, a highly cross‐linked network experienced fragmentation as a result of cleavages of these bonds.

The degraded products were subsequently processed into fine powder through grinding and drying (Figure 2c). Notably, by mixing 1 g of degraded powder with ≈5 g of our pristine epoxy resin (refer to Section S1.7, Supporting Information) and curing following the same procedure, we obtained the post‐cycled EP with impact strength and flexural strength comparable to the original (Figure 2d). Despite the cleavable bonds being introduced, we note that in thermosetting materials with only adapting additively crosslinked cleavable units, theoretically, it is difficult in practice to realize entirely or closed‐loop recycling of monomers once all of the crosslinks are formed.^[^ ^64^, ^65^, ^66^ ^]^ In this system, although the native EP thermoset also contains ester dynamic bonds, it remains non‐degradable or slowly degradable compared to the vitrimer. Thus, both the cleavable units and topological crosslinks control the degradation.

Regarding the recovery uses (Figure 2g–i), carbon fiber composites have been explored for high‐performance applications, while the costly embedded carbon fibers (CFs) cannot be retrieved from bulk materials.^[^ ^67^, ^68^ ^]^ In this implementation, when carbon fiber fabrics were embedded into HDCNs to fabricate a composite, we were able to quantitatively recover the CFs while effectively separating the end‐of‐use epoxy powders from the composite. The Raman spectrum of the recovered fibers shows a carbon peak closely resembling those of the native fibers, indicating minimal surface impact on the carbon fibers (Figure 2j). These results hint at promising opportunities for the cyclic utilization of end‐of‐use epoxy resins and composites.

### 2.3 Reconstructed HDCNs Enable Reconfigurable Elastic Vitrimer

**Figure 3:**
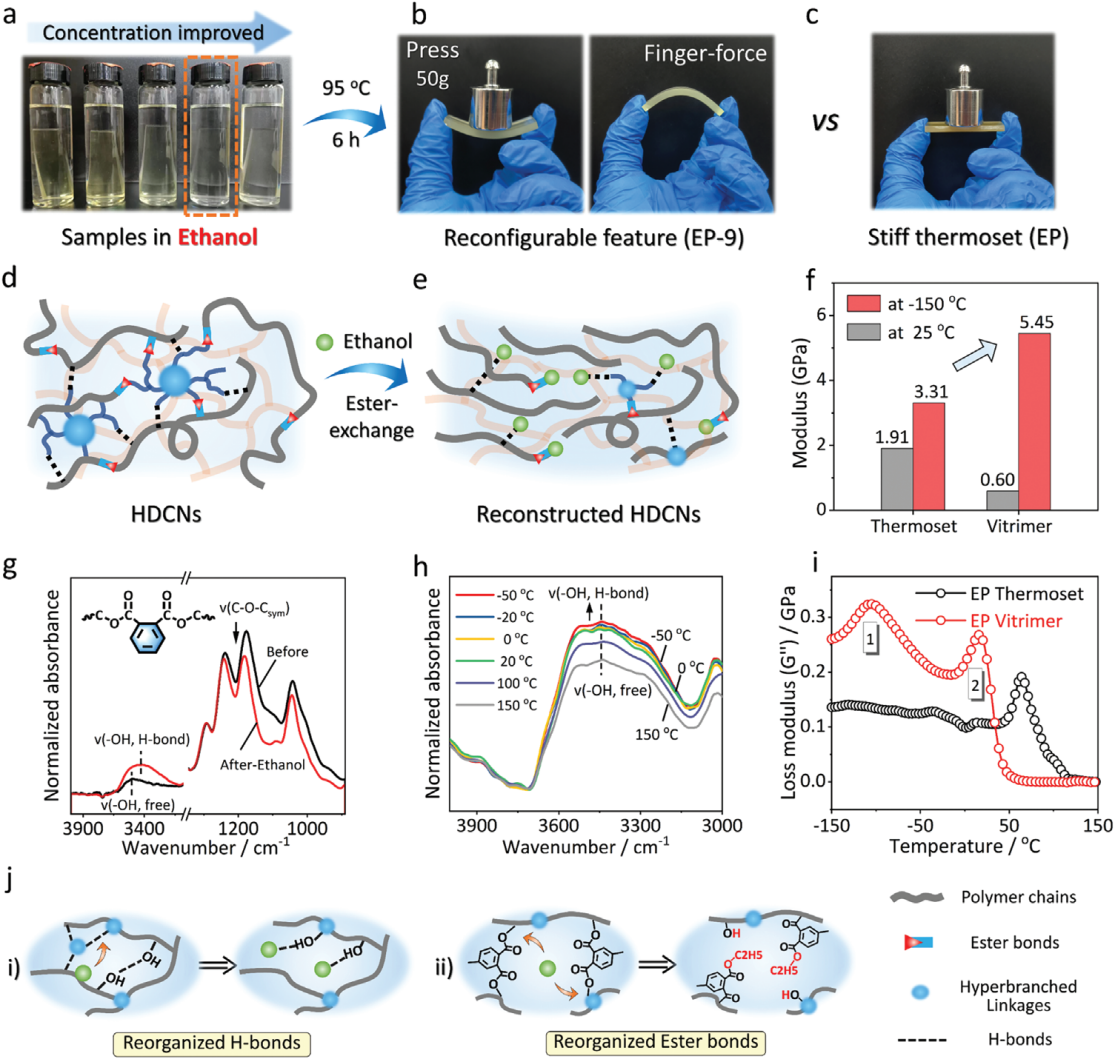
Reconstructed HDCNs enable a reconfigurable yet tough vitrimer. a) Samples immersed in ethanol for the reconstruction of HDCNs. b,c) digital photographs of the elastomeric vitrimer (EP‐9) versus rigid thermoset (EP). d,e) ethanol‐induced reconstruction of HDCNs from ester‐exchange. f) *E’* showing an ultrahigh flexural modulus at −150 °C for low‐temperature application. g) FTIR spectra of the vitrimer before‐ and after‐ ethanol treatment. h) 2D temperature‐dependent FTIR upon heating from −50 to 150 °C. i) loss modulus (*G’’*) corresponds to double‐relaxation behaviors. J) Possible HDCNs reconstruction mechanism: reorganized H‐bonds and dynamic ester bonds.

Next, by a chance following‐up, we note that when the samples were immersed in ethanol following a heating (95 °C, 6 h), the vitrimer sample (EP‐9) via forming HDCNs displayed elastomeric‐like feature upon cooling to room temperature, which allowed for a casual deformation and recovery as well (see Movie S1, Supporting Information; **Figure** **3**a,b), whereas the native thermoset (EP) still remains its rigid stiffness (Figure 3c). In our further study, we denoted EP‐9 as an example with suitable elasticity compared to EP‐12. Such transformation in elastomeric characteristics is exciting considering the inherent highly crosslinked nature of its bulk thermosets, especially for industrial epoxy commodities. This suggests that hyperbranched cross‐linking may alter the crosslinking mode of the thermoset network. We coined this type of vitrimer as “reconfigurable” vitrimers,^[^ ^16^, ^49^ ^]^ that is, a thermosetting network – whose crosslinked structure should be permanent – but can be reconstructed through solvent‐assisted reconfiguration. As illustrated in Figure 3d,e, we reasoned the ester‐exchange driving the reconstruction of HDCNs,^[^ ^69^, ^70^ ^]^ whereby the ester bonds and hydrogen bonds can be rearranged since the ethanol serves as a single ─OH blocking molecule to substitute the ─OH site from those of hyperbranched crosslinks. The reorganized structure (Figure 3j) is corroborated by FT‐IR before‐ and after‐ethanol, temperature‐dependent IR, and dynamic thermomechanometry (Figure 3g–i). In particular, Figure 3g demonstrates a blueshift in the hydroxyl stretching vibration at 3400 cm^−1^, along with an increased width and intensity, indicating the transition of hydroxyl groups from a “free” state to an “associated” state. Furthermore, the reduction in peak intensity of C─O─C_sym_ verifies the occurrence of ester exchange in HDCNs since symmetric ether structures (C─O─C_sym_, 1200–1150 cm^−1^) typically exhibit an increase in peak intensity. Additional evidence from model reactions (Section S1.8, Supporting Information)^[^ ^50^, ^53^ ^]^ and the XPS survey (Section S2.8, Supporting Information) further supports the ester exchange should be the main mechanism to drive the reconfigurable behavior of the material. Ultimately, the reconstruction of HDCNs introduces polymer chain mobility and reduces the crosslinked structure, leading to the transformation in material nature.

To further investigate the network dynamics of HDCNs, dynamic thermomechanometry was investigated over a wide temperature from −150 to 150 °C (Figure 3i; Figure S23, Supporting Information) to record flexural modulus at three‐point bending mode. As the temperature decreases, the loss modulus exhibits a double‐relaxation of heat dissipation when the material is deformed. The lower (mark 1) relaxation involves a glass‐to‐elastic transition that unfreezes the side chains and groups, resulting in a rapid increase in flexural storage modulus from 3.2 to 5.4 GPa (Figure S23a, Supporting Information). This increase is attributed to the ethyl ester (forming by ethanol exchange) occupying the initial crosslinked site of carbonic ester for network reconstruction (Figure 3j). The second relaxation (mark 2) signifies an elastic‐to‐liquid transition as the vitrimer network flows and relaxes due to dynamic bond exchange. Real‐temperature IR analysis confirms the defreezing temperature of H‐bonds in close proximity to this transition, as indicated by a significant increase in the normalized intensity of hydroxyl groups, shifting from the “associated” state to the “free” state upon temperature elevation from 0 °C (Figure 3h),^[^ ^71^ ^]^ while the intensity remains almost unchanged from −50 to 0 °C.

In this term, the reconstructed HDCNs display a stronger H‐bonding crosslinked network compared with that of native EP at lower temperature areas, where the polymer segments are frozen and tightly constrained by H‐bonding.^[^ ^72^ ^]^ Consequently, we observe a significant increase in *G’* with decreasing temperature (Figure S23a, Supporting Information), recording as high as 5.45 GPa at −150 °C. This represents an exceptionally high flexural modulus for polymeric materials and approximately twice that of native EP under the same condition. The exceptional modulus at ultra‐low temperatures empowers this polymeric material with supernormal applications in astrospace material, superconducting energy storage, and medical equipment.

### 2.4 General Properties and Multifunctional Performance of HDCNs

**Figure 4:**
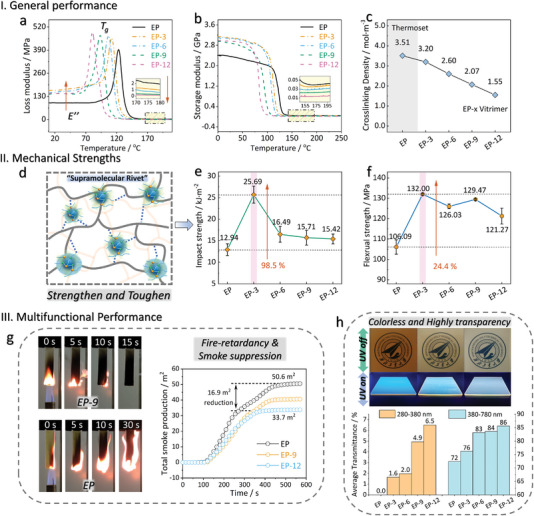
General and multifunctional performance of material. DMA determination for a) temperature‐dependent loss modulus and b) storage modulus of materials. c) Crosslinking density showing the reduction in network density due to network topology. d) Simultaneously strengthening and toughening effect through HDCNs. e,f) Impact toughness and flexural strength improvement. g) Self‐extinguishing performance and reduced smoke production for fire‐retardancy. h) Digital photos of typical samples (EP, EP‐9, EP‐12 from left to right) and average transmittance in visible (220–380 nm) and UV region (380–780 nm).

**Figure** **4** provides a comprehensive analysis of the multifaceted performance of the material in harnessing the advantages of HDCNs. First, the glass transition temperature (*T*_g_, Figure S21a and Table S6, Supporting Information) of EP, EP‐3, EP‐6, EP‐9, and EP‐12 are 131, 121, 118, 104, and 94 ^°^C, respectively, indicating they are glassy polymers at room temperature. The slight decrease in *T*_g_ can be attributed to the amorphous structure of HBPPB.^[^ ^38^ ^]^ Upon crosslinking a hyperbranched macromolecular, network topologies are physically expanded.^[^ ^73^, ^74^ ^]^ However, the heat loss of chains motion is intensified below *T*_g_ due to increased friction and constraints imposed by the HBPPB within the network (increased loss modulus (Figure 4a). Conversely, above *T*_g_ and *T*_v_, the friction between epoxy chains with HBPPB is reduced due to dynamic bond exchange, and the network flows more easily thus existing a reduced loss modulus. Note that inserting poorly rigid chains often compromises the modulus and strength. Remarkably, the vitrimer, upon introducing an aliphatic hyperbranched crosslinker, demonstrates an even higher storage modulus (*E’*) compared to that of native EP (Figure 4b). Specifically, the addition of 3% HBPPB elevates *E’* from 2.38 GPa of native EP up to 3.15 GPa (Figure S21b, Supporting Information), followed by a decline with further increasing HBPPB, which substantiates the reinforcement effect of HDCNs. This enhancement can be attributed to that the hyperbranched structure acts as a “supramolecular rivet” to facilitate a robust supramolecular/epoxy interpenetrating network through H‐bonds and covalent bonds (Figure 4d).^[^ ^75^, ^76^ ^]^ The branching molecular topology enables an expanded network interspace, resulting in a reduced network crosslinking density (Figure 4c), as elaborated in Section S2.1 (Supporting Information). Consequently, the hyperbranched crosslinker holds significant promise as a topological crosslinking tool for programming low‐crosslinking yet high‐strength polymers.

Measurements of impact toughness, flexural strength, and tensile strength were carried out to facilitate a comprehensive understanding of mechanical performance (Figure 4e,f; details in Section S2.6, Supporting Information). The high crosslinking density and rigid polymer chains render thermosets susceptible to impact failure, resulting in a microscopic “river‐like” brittle fracture (Figure S30, Supporting Information) with 12.94 kJ m^−2^ of impact strength. However, EP‐3 shows a superior increase (98.5%) from 12.94 up to 25.69 kJ m^−2^, accompanied by obvious “dimple‐fracture” features that promote energy absorption. Thought‐out relevant works regarding hyperbranched polymer‐reinforced thermosets, a supramolecular network‐induced energy dissipation mechanism could be supposed.^[^ ^77^, ^78^ ^]^ The network topologies furnish cross‐linked polymers with enhanced properties without altering their chemical composition.^[^ ^79^ ^]^ It is worth noting that the increase in impact strength surpasses that of flexural strength, which can be attributed to the presence of flexible aliphatic chains that mitigate the network stiffness. Both the flexural and tensile strength show a consistent and noteworthy improvement from 106.1 up to 132 MPa, and 52.1 up to 81.2 MPa, respectively (Table S7, Supporting Information). Notably, the tensile strain‐stress curves indicate that vitrimers are more stretchable than that of native thermoset epoxy (Figure S31, Supporting Information). This finding holds great promise for the fabrication of carbon fiber reinforced polymers (CFRPs), where the stress should primarily reside in the fibers rather than the resins. This attribute ensures the exceptional mechanical strength of CFRPs (Section S2.6.3, Supporting Information).

In this contribution, both the strength and modulus depend on a tightly crosslinked network. The HBPPB, which terminates abundant ─OH groups, acts as a “rivet” within the network through covalent bonding and non‐covalent H‐bonding. The observed increase in *E’* is also in agreement with the flexural results. Unfortunately, the optimal mechanical system does not maintain a consistent HBPPB concentration with the degradable and recyclable sample. Despite overloading HBPPB causing deteriorated mechanical strength, it still outperforms native EP. The reduction in impact and flexural strength can be attributed to the local agglomeration of hyperbranched polymers.^[^ ^45^, ^46^ ^]^ Supplementary thermal and thermomechanical parameters are provided in Table S6 (Supporting Information). The vitrimers exhibit improved *T*_max_ values and char yields, indicating superior thermal stability of HDCNs, which is primarily attributed to the presence of the inert boron component in the system.

We next sought to probe the functional performance of materials. Good fire safety is a crucial consideration for real‐world applications of polymeric materials.^[^ ^80^, ^81^ ^]^ Overall, our results demonstrate that the vitrimer sample exhibits superior fire safety performance compared to the native EP. Specifically, the vitrimer samples, EP‐9 and EP‐12, exhibit reduced thermal hazards and smoke production, along with increased resistance to ignition (higher limit oxygen index), and self‐extinguishing properties in an air atmosphere (Figure 4g). A comprehensive investigation was conducted through a full‐scale study of the fire‐retardant performance (Section S2.10 and Figure S41, Supporting Information). The cone calorimeter test, which simulates a real fire scenario, provided crucial parameters to elucidate the flame‐retardant effect and mechanism.^[^ ^82^ ^]^ Additionally, XPS analysis and Raman spectra (Figure S42, Supporting Information) revealed the condensed fire‐retardant actions. The HDCNs constructed in our materials accumulate two types of flame‐retardant elements (phosphorus and boron) within the hyperbranched backbone for synergistic fire‐retarding action, ensuring the fire safety of the material.

One of the facts is that the materials, specifically EP‐9 and EP‐12, exhibit significantly superior transparency and lighter coloration than their native thermoset. The transmittance of materials was measured using UV–vis spectrophotometer (See Figure S40, Supporting Information). The average transmittance in both 280–380 nm (UV region) and 380–780 nm (visible region) was acquired in Figure 4h, showing significant increases in transmittance to both UV and visible light. The exceptional transparency of these materials can be attributed to the unique construction of HDCNs and the optical properties of HBPPB. Excitingly, this type of hyperbranched polymer has been reported as a class of non‐traditional aggregation‐induced emission luminogens (AIEgens),^[^ ^83^, ^84^ ^]^ posing a promising pathway toward understanding the polymer crosslinking via fluorescent visualization methods. Besides, the exceptional transparency and lightness impart this polymeric material high added‐values for application in packaging materials, photoelectric materials, and flexible electronics.^[^ ^85^, ^86^ ^]^

## 3 Conclusion

In summary, we concept a hyperbranched dynamic crosslinked network (HDCNs) featuring multi‐dynamic bonds and topological crosslinking network architecture. Such a unique structure not only allows the penetration of solvent molecules for dynamic responsiveness, but also integrates exceptional mechanical strength, high transparency, and fire‐retardant functions into a single material. Through the construction of HDCNs, this work engineers a commercial petroleum‐based epoxy thermoset into a degradable and reconfigurable vitrimer by the breakages of dynamic units and the reconstruction of dynamic networks. The vitrimer showcases mild solvent degradation at room‐temperature and powder reprocessable performance, enabling the recovery of carbon fiber and resin powder from composite. More remarkably, we have made an exciting discovery that HDCNs display reconfigurable behavior via ester‐exchange triggered by ethanol, resulting in a flexible elastomer at 25 °C and a supra‐high flexural modulus of 5.45 GPa at ultralow temperature (−150 °C), thus offering the potential for extraordinary applications. The utilization of HDCNs as a building strategy opens up many inspirations toward designing and customizing sustainable polymeric materials, while also uncovering their fascinating material nature.

## Conflict of Interest

The authors declare no conflict of interest.

## Supporting information

Supporting Information

Supplemental Movie 1

## Data Availability

The data that support the findings of this study are available from the corresponding author upon reasonable request.
