# Supplementary material for: Hyperbranched Dynamic Crosslinking Networks Enable Degradable, Reconfigurable, and Multifunctional Epoxy Vitrimer
Source: Adv Sci (Weinh). 2023 Nov 7;11(2):2306350. doi: 10.1002/advs.202306350 (PMC10787098; doi:10.1002/advs.202306350)
Supplement: Supplementary file 1 — Supporting Information [file ADVS-11-2306350-s002.pdf]

ADVANCED SCIENCE
Open Access

Supporting Information

for *Adv. Sci.*, DOI 10.1002/advs.202306350

Hyperbranched Dynamic Crosslinking Networks Enable Degradable, Reconfigurable, and Multifunctional Epoxy Vitrimer

Yuanbo Zhang, Hongxia Yan, Ruizhi Yu, Junshan Yuan, Kaiming Yang, Rui Liu, Yanyun He, Weixu Feng and Wei Tian**

Supporting Information

Hyperbranched Dynamic Crosslinking Networks Enable Degradable, Reconfigurable and Multifunctional Epoxy Vitrimer

Yuanbo Zhang, Hongxia Yan, Ruizhi Yu, Junshan Yuan, Kaiming Yang, Rui Liu, Yanyun He, Weixu Feng, and Wei Tian**

S1. Materials and Methods

S1.1. Materials

The chemicals used in this study were sourced from reputable suppliers. 1,3-Propanediol (PDO, AR), tributyl borate (TBB, AR), triethyl phosphate (TEP, AR), methylboronic acid (MBA, AR), 1-butanol (AR), and potassium bromide (KBr, SP) were purchased from Macklin Biochemical Co., Ltd. Methylphosphonic acid (MPA, AR) was purchased from Zhengzhou Huiju Chemical Co., Ltd. All solvents employed analytical reagents, including ethanol (EtOH, AR), dimethylacetamide (DMAc, AR), dimethylformamide (DMF, AR), and etc, and were obtained from Guanghua Sci-Tech Co., Ltd. Chloroform-*d* ($CDCl_3$) and dimethyl sulfoxide-*d6* (DMSO-d_6) were purchased from Shanghai Acmec Biochemical Co., Ltd. No further purification of the chemicals was necessary. The epoxy resin employed in this study was based on a commercially-used bisphenol A diglycidyl ether (DGEBA, E51), and was obtained from Guangzhou Suixin Fine Chemical Co., Ltd. Methyl Tetrahydrophthalic Anhydride (MTHPA, mixture of isomers) was purchased from Qihua Chemical Co., Ltd., while tris(dimethylaminomethyl)phenol (DMP-30) was sourced from Macklin Biochemical Co., Ltd. The carbon fiber fabric (T300, K12) was purchased from Changzhou Saweilang Composite Material Co., LTD. The casting mold was fabricated in-house.

S1.2. General characterizations

The Nicolet Is50 FT-IR spectrometer (U.S.A) equipped with an attenuated total reflectance (ATR) model was employed to record Fourier transform infrared spectra (FT-IR). In-situ FT-IR measurements were conducted over a temperature range of -50 ^oC to 150 ^oC using a Thermo IS50 instrument. Nuclear magnetic resonance spectroscopy of ^1H, ^{13}C and ^{31}P (^1H-NMR, ^{13}C-NMR, ^{31}P-NMR) was performed using a Bruker Avance 400 MHz spectrometer. ^IH-NMR

analysis of small molecular model reaction was performed on a Bruker Advance Neo 500 MHz spectrometer. The concentration of the -OH group was determined by titrimetric test, in accordance with GB/T 7383-2007 (Chinese standard). The molecular weight information was determined by Gel Permeation Chromatography-Eighteen Angle Laser Scatterer (GPC-MALLs, Waters), equipped with a DAWN HELEOS II system (LS) differential refraction detector (dRI) with tetrahydrofuran (THF, SP) as mobile phase.

The mechanical flexural and impact strengths were measured using an electro-mechanical tester in accordance with GB/T 2567-2008 (Chinese standard). The sample dimensions were 80 × 15 × 4 mm^3 (flexural strength) and 80 × 10 × 4 mm^3 (impact strength). Dynamic thermomechanical analysis (DMA, NETZSCH DMA 242E) was performed using a three-point bending model at the frequency of 1 Hz. The storage and loss modulus were continuously recorded from 50 ^oC to 300 ^oC, and the samples were monitored at a wider temperature range of -150 ^oC to 200 ^oC after solvent treatment. Differential scanning calorimetry was conducted under N_2 atmosphere using a Mettler-Toledo DSC3. The rheological measurements of the samples were performed on a stress-controlled rheometer (ThermoFisher Mars) using a 25 mm parallel plate under isothermal time-sweep mode at 150 ^oC with a constant frequency of 1 Hz. Thermogravimetric analysis was performed under nitrogen atmosphere using a STA 449F3 thermal analyzer with a heating rate of 15 $°C\cdot min^{-1}$. The impact fracture surface was photographed using a Scanning Electron Microscope equipped with a tungsten filament gun (TESCAN VEGA 3 LMH), while Raman spectra were obtained using an Alpha300R spectroscopy equipped with a 532 nm TEM00 laser.

The fire-retardant performance was evaluated using a Cone calorimeter test at a heat flux of 35 kW/m^2, according to ISO 5660 standard. The sample dimensions were 100 × 100 × 4 mm^3. The char residue after the cone test was examined using a field emission Scanning Electron Microscope (FEI Verios G4). The limited oxygen index (LOI) was measured using samples of size 80 × 10 × 4 mm^3, in accordance with GB/T 2406.2-2009 (Chinese standard). The UL 94 vertical burning test was carried out according to ASTM D3801, with specimen dimensions of 125 × 13 × 4 mm^3. The transparency was measured using a UV-vis spectrophotometer (Hitachi U-3900), and the average transmittance over the wavelength range was obtained by integrating the measured data.

S1.3. Synthesis details and structural information for HBPPB

a. Synthesis details of HBPPB

In this study, a hyperbranched phosphate/borate hybrid polymer (HBPPB) was synthesized via a one-pot A_2+B_3+C_3 transesterification. The resulting polymer, as depicted in **Figure S1**, shows a representative model of the hyperbranched ternary structure. In such structure, the macromolecular backbone is randomly linked with phosphorus and boron sites. The feed ratio was strictly controlled in accordance to Flory-Carothers law to avoid over-crosslinking. The surplus PDO served as the donor monomer to terminate abundant hydroxyl groups in resulting polymer.

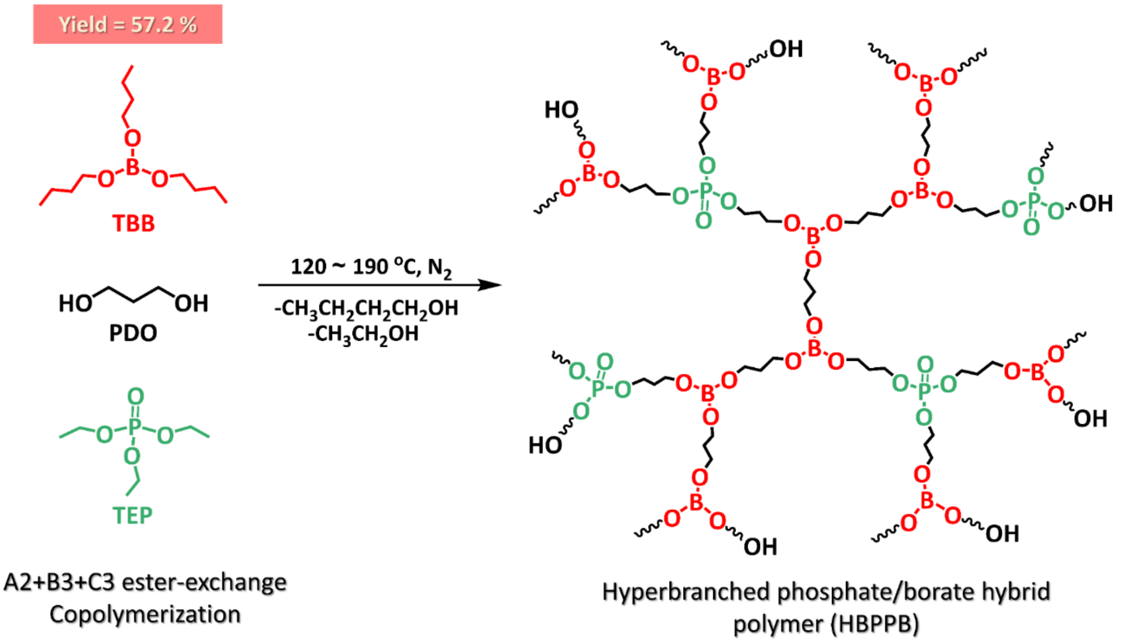

Figure S1 Synthesis of HBPPB

Specifically, 0.35 mol of 1,3-propanediol (PDO) was mixed with 0.08 mol tributyl borate (TBB) and 0.08 mol of triethyl phosphate (TEP) in a three-necked flask, equipped with a mechanical stirrer, an N_2 inlet, and a condenser. The reaction process comprised of three stages, each occurring within a different temperature range. Throughout the polymerization, the by-product distillate can be continuously separated from reaction system by the condenser to push the reaction forward, and the reaction distillate was collected for FT-IR detection. Firstly, the mixture was heated from 120 °C to 160 °C at a heating rate of 5 °C h^{-1}. Subsequently, the temperature was raised from to 180 °C for maintaining 2 hours. Finally, the elevated temperature was allowed for 190 °C and maintained until no more distillate generated. The

product was purified through dialysis to remove redundant small molecules, followed by pressure-reduced drying. This yielded a yellowish liquid product, namely HBPPB, with a yield of approximately 57.2% and molecular weight of Mn = 64.2 kDa (**Table S1**), calculated based on the mass of the final product relative to the mass of initial feed.

b. Structural information of HBPPB

The molecular structure of HBPPB was investigated using several techniques including by fourier transform infrared (FT-IR) spectroscopy, ^{1}H, ^{13}C and ^{31}P nuclear magnetic resonance (NMR) spectroscopy. Initially, FT-IR was employed to determine the functional groups in the monomers (TBB, TEP and PDO) and the HBPPB (**Figure S2a**). The IR profiles of TBB and TEP showed similar peaks at 1050 cm^{-1}, corresponding to similar ester groups of B-O-C and P-O-C, respectively, which also appeared in the spectrum of HBPPB. The peak at 1450 cm^{-1} observed in PDO, attributed to the stretching vibration of C-OH, was also present in the spectrum of HBPPB. This indicated that the chemical structure of HBPPB contained B-O/P-O groups as well as C-OH and/or C-O-B and/or C-O-P stretching vibrations from PDO, suggesting the occurrence of the reaction.[1] The spectrum of HBPPB also exhibited abundant hydroxyl groups (-OH) in the range of 3000 to 3500 cm^{-1}, with a peak at 2850 cm^{-1} to 3000 cm^{-1} assigned to the methylene group.

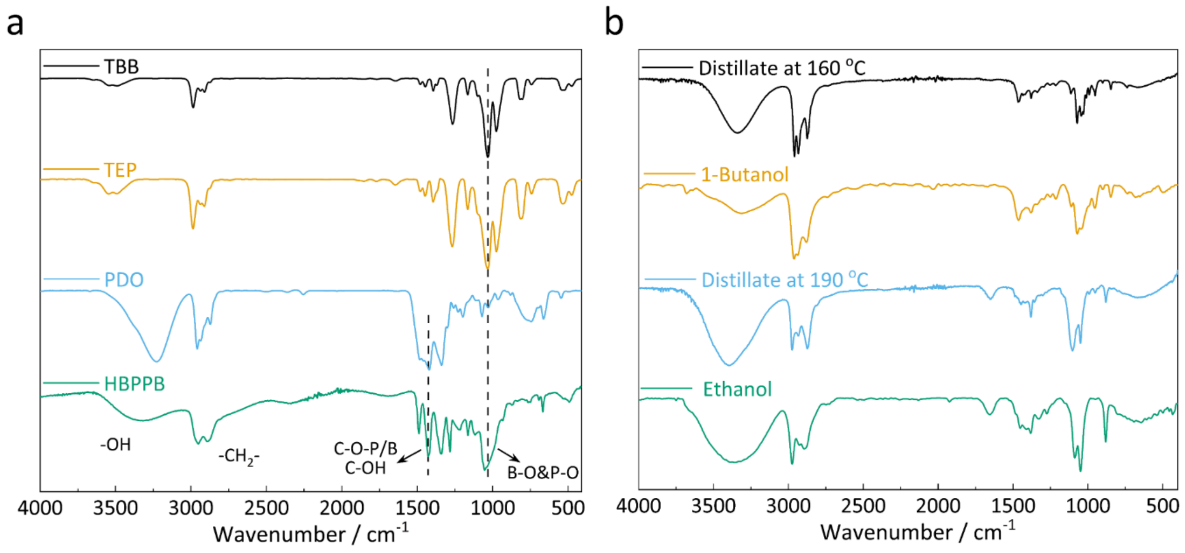

Figure S2 FTIR spectra of a) the monomers and the as-synthesized HBPPB, b) reaction distillate comparing with standard 1-butanol and ethanol

Further evidence of the reaction was obtained from the IR profiles of the distillate collected at different temperatures (160°C and 190°C). In **Figure S2b**, the distillate at 160°C primarily consisted of 1-butanol, while the distillate at 190°C mainly contained ethanol. This indicated that transesterification had occurred between TBB and PDO before the reaction with TEP.

The ^{1}H-NMR and ^{13}C-NMR spectra of TBB, TEP, PDO, and HBPPB provided additional structural evidence (**Figure S3**). DMSO-*d*$_6$ was selected for TEP and HBPPB, while $CDCl_3$ were chosen for TBB and PDO. It's is worth noting that the proton and carbon from H2 and C2 in PDO are separated into different modes in the spectrum of HBPPB, corresponding to the various link mode and site of boron and phosphorus within hyperbranched structure, indicating the proceeded polymerization.

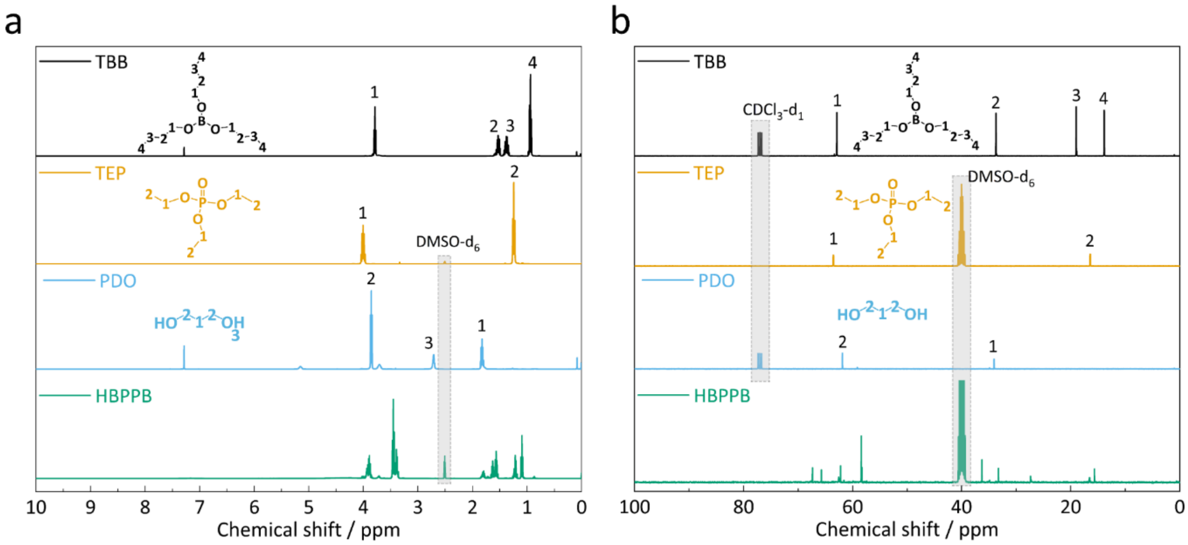

Figure S3 a) ^{1}H-NMR and b) ^{13}C-NMR of monomers and as-synthesized HBPPB

The ^{1}H-NMR details of HBPPB, as shown in **Figure S4**, provides further assignment of the proton signals and their corresponding chemical shifts. The signal at 3.91 ppm (H6&H8) is associated to the methylene proton of P-O-CH_2-CH_2-$C\underline{H}_2$-OH and B-O-CH_2-CH_2-$C\underline{H}_2$-OH, respectively. The close chemical shifts at 3.45 ppm (H2) and 3.39 ppm (H4) correspond to the methylene protons of B-O-CH_2- and P-O-CH_2-, respectively, which are close to the chemical shift of H1 in TBB and TEP. The signals at 1.09 ppm, 1.22 ppm, 1.56 ppm, 1.62 ppm, 1.80 ppm are associated with B-O-CH_2-$C\underline{H}_2$-CH_2-O-B (H1), B-O-CH_2-$C\underline{H}_2$-CH_2-O-P (H3), P-O-CH_2-$C\underline{H}_2$-CH_2-OH (H5), B-O-CH_2-$C\underline{H}_2$-CH_2-OH (H7), P-O-CH_2-$C\underline{H}_2$-CH_2-O-P(H9), respectively. Additionally, a broad signal in the range of 4.1-4.6 ppm corresponds to the active proton of the hydroxyl group.[2]

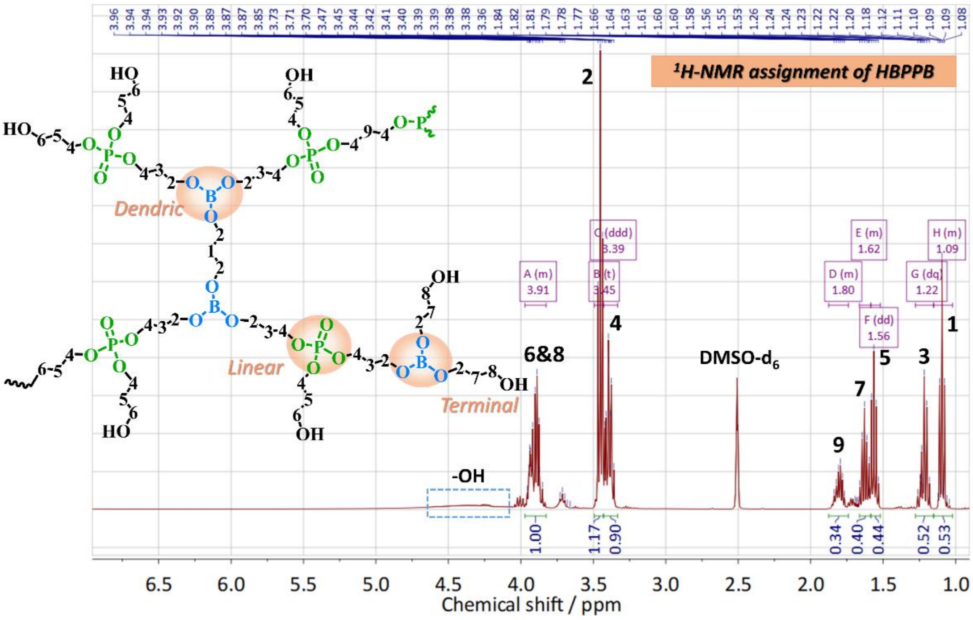

Figure S4 The ^{1}H-NMR assignment of HBPPB

The ^{13}C-NMR of HBPPB, presented in **Figure S5**, assigns the carbon signals based on the different functional groups in the polymer. As a result of the polymerization, the carbons from PDO are divided into four types (C2, C4, C6, C8), which are associated to the carbons in B-O-$\underline{C}H_2$-, P-O-$\underline{C}H_2$-, P-O-CH_2-CH_2-$\underline{C}H_2$-OH and B-O-CH_2-CH_2-$\underline{C}H_2$-OH, respectively. The methylene carbon of C1, C3, C5, C7, C9 are correlated to P-O-CH_2-CH_2-$\underline{C}H_2$-OH, P-O-CH_2-CH_2-$\underline{C}H_2$-OH, P-O-CH_2-CH_2-$\underline{C}H_2$-OH, P-O-CH_2-CH_2-$\underline{C}H_2$-OH, P-O-CH_2-CH_2-$\underline{C}H_2$-OH, providing evidence for the ternary structure of HBPPB.

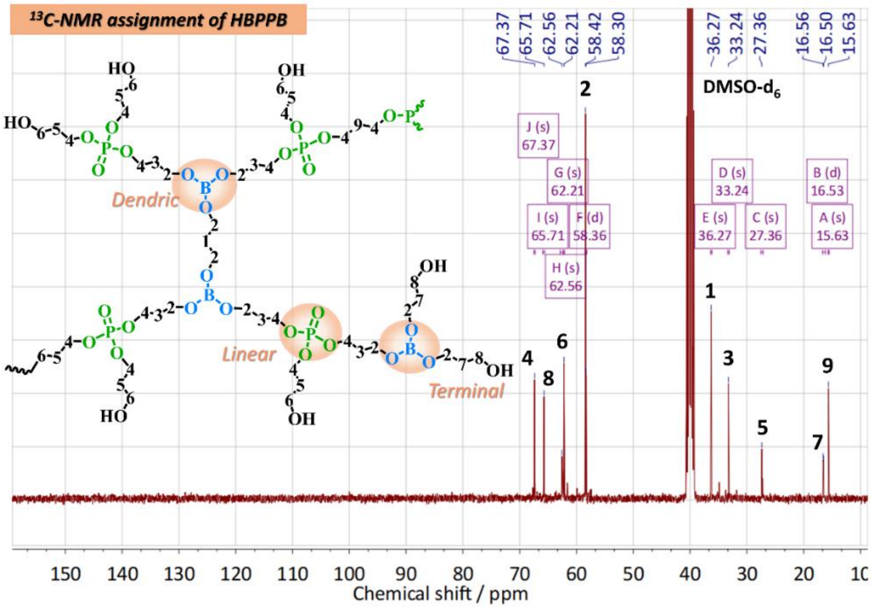

Figure S5 The ^{31}C-NMR assignment of TEP and HBPPB

Generally, the chemical shifts of tetra-coordinated phosphorus(V)-oxygen compounds range from about 0 ppm to -30 ppm, including phosphate and their compounds in ester state. After polymerization, a small offset from -0.9 ppm to -1.2 ppm is recognized in the ^{31}P-NMR spectrum of HBPPB, demonstrating that HBPPB still retains the phosphate structure (**Figure S6**). Additionally, the phosphorus signal in HBPPB splits into five spikes in a closer range, corresponding to the phosphate units at different sites adjacent to boron sites within the hyperbranched structure.[3]

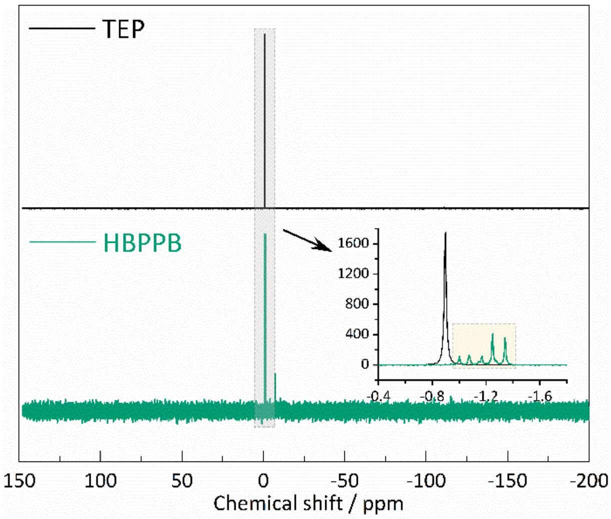

Figure S6 The ^{31}P-NMR spectra of TEP and HBPPB

The gel permeation chromatography gives the molecular weight information of HBPPB (**Table S1**). The Gel Permeation Chromatography-Eighteen Angle Laser Scatterer (GPC-MALLs) equipped with DAWN HELEOS II system and differential refraction detector, with tetrahydrofuran (THF, SP) as mobile phase. The number-average molecular weight (Mn) was determined as 64.2 kDa, proving the macromolecular structure of HBPPB.

Table S1 Molecular weight information of HBPPB

Sample	M_n (kDa)	M_w (kDa)	M_z (kDa)	PDI (M_w/M_n)
HBPPB	64.2	157.4	395.4	2.45

c. Titration experiment for hydroxyl concentration of HBPPB

The concentration of hydroxyl group within HBPPB was determined using a titration experiment following the procedure outlined in GB/T 7383-2007 (Chinese standard). Firstly, approximately 1.00 g of HBPPB was mixed with 25 ml phthalic anhydride pyridine solution

(140.00 g to 1 L pyridine). The mixture was heated at 115 °C for 60 minutes under a condensing reflux. Next, 4 ~ 5 drops of phenolphthalein indicator were added, and the solution was titrated with 0.5 M sodium hydroxide solution (repeat 3 times) until the pink solution maintaining for more than 15 s. A control trial tset without HBPPB was carried out for three times. The average $Q_{(OH)}$ can be calculated using **Equation (1)**[4], as given in **Table S2**.

$$Q_{(OH)} = \frac{c \times (V_0 - V_1)}{m_0} \quad (1)$$

Where c is the concentration of sodium hydroxide solution (0.50 mol·L^{-1}); V_0 and V_1 are the volume of sodium hydroxide solution consumed by controlled trial and HBPPB samples, respectively; m_0 is the mass of HBPPB.

Table S2 Titration experiment records for the -OH concentration of HBPPB

Test number	c (mol·L^{-1})	V_0 (ml)	V_1 (ml)	m (g)	$Q_{(OH)}$ (mol g^{-1})
Control 1	0.50	87.50	—	—	—
Control 2	0.50	86.50	—	—	—
Control 3	0.50	88.00	—	—	—
Average	0.50	87.33	—	—	—
Test 1	0.50	87.33	70.50	1.007	8.36×10^{-3}
Test 2	0.50	87.33	71.40	1.005	7.92×10^{-3}
Test 3	0.50	87.33	71.80	1.017	7.63×10^{-3}
Average	—	—	—	—	7.97×10^{-3}

S1.4. Synthesis details and characterizations for HBPB and HBPP

In parallel, two comparable hyperbranched structures, namely hyperbranched polyborate (HBPB) and hyperbranched polyphosphate (HBPP), were synthesized as controls, each featuring single dynamic bonds of BO_3 and PO_3 (**Figure S7**). The synthesis followed the same molar ratio as that of HBPPB. For the synthesis of HBPB, 0.16 mol TBB was mixed with 0.35 mol of PDO in a 100 mL three-necked flask equipped with a mechanical stirrer, an N_2 gas inlet and a thermometer. The mixture was stirred and heated from 120 °C to 190 °C at a heating rate of 5 °C·h^{-1} with continuous N_2 blowing-in. The by-product was separated from reaction system by a condenser until no more distillate generating. The product was collected and dialyzed to remove small molecular substances. For the synthesis of HBPP, 0.35 mol of PDO was mixed with 0.16 mol of TEP using the same reactors. The mixture was heated from 120 °C to 190 °C under the heating rate of 5 °C·h^{-1}. Until no more distillate generating, the reaction product was collected and dialyzed. Finally, the two dialysate liquids were rotary evaporated at 45 °C and dried in a 60 °C vacuum for 6 h, yielding viscous liquid product, denoted as HBPB and HBPP.

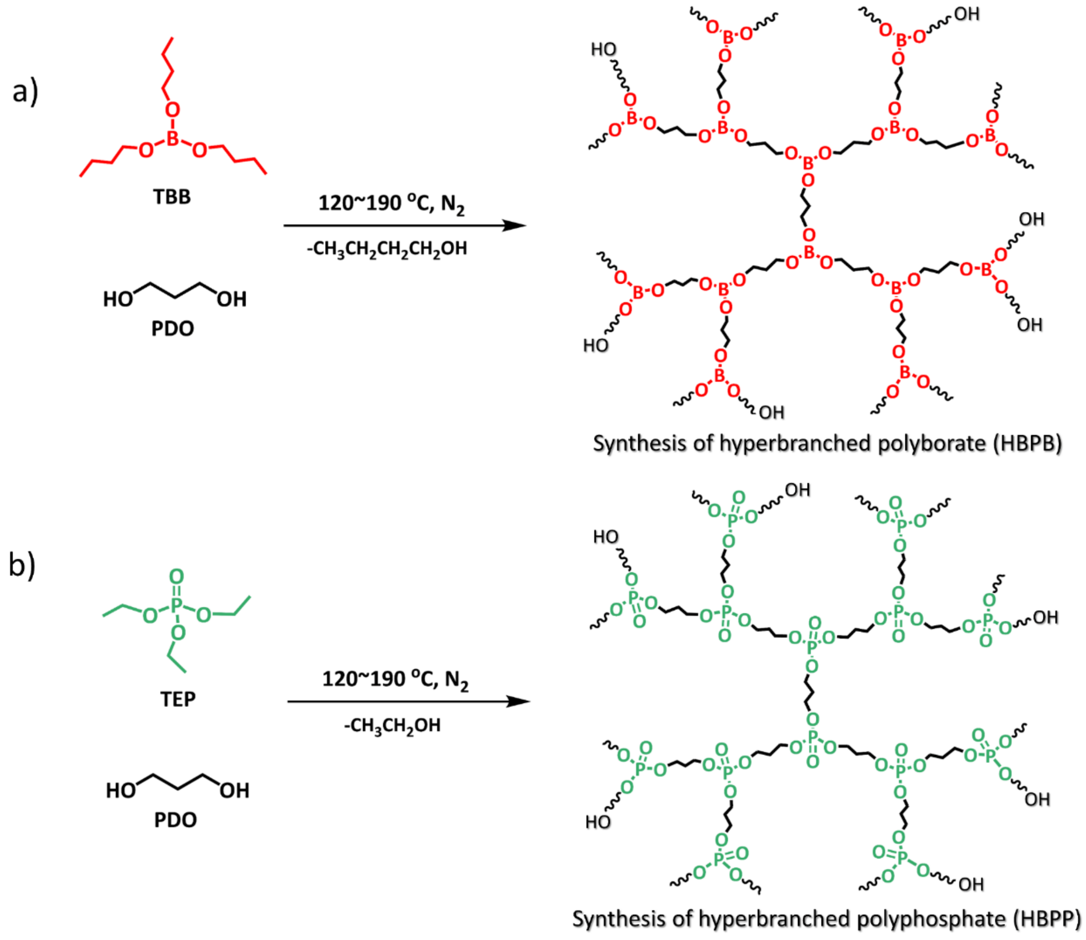

Figure S7 Synthesis route of HBPB and HBPP

The structure evidence of HBPB is referred to the previous study of our group.[5] Since the poor compatibility between HBPP with epoxy matrix, the structural information of HBPP is not discussed in the current context, and only exploratory experiments are carried out.

S1.5. Synthesis details and characterizations for LPPB

Concurrently, a linear poly-phosphate/borate hybrid (LPPB) was synthesized to provide a point of comparison with the hyperbranched structure. Two monomers including a methylboronic acid (MBA) and a methylphosphonic acid (MPA) were selected as substitutes for the ternary monomers in HBPPB, allowing for esterification with PDO.

In brief, the reaction followed the feed ratio of 0.35 mol of PDO, 0.12 mol of MBA and 0.12 mol of MPA, thereby ensuring an equivalent molar amount of dynamic units compared to HBPPB. Dehydration catalyst in the form of 0.05 g of p-toluenesulfonic acid was added. Since the synthesis relied on the dominance of kinetic route for polymerization rather than intermolecular cyclization, the reaction was therefore conducted at a relatively low temperature (120 ^{o}C) for long-time polymerization until no more distillate generated. The distillate was indentified as water since an anhydrous copper sulfate quickly turns blue, indicating the as-proceeded polymerization. The reaction product was initially purified using a dialysis bag (D2000) to eliminate the small molecules, yielding a yellowish liquid product with a yield of approximately 70.6%.

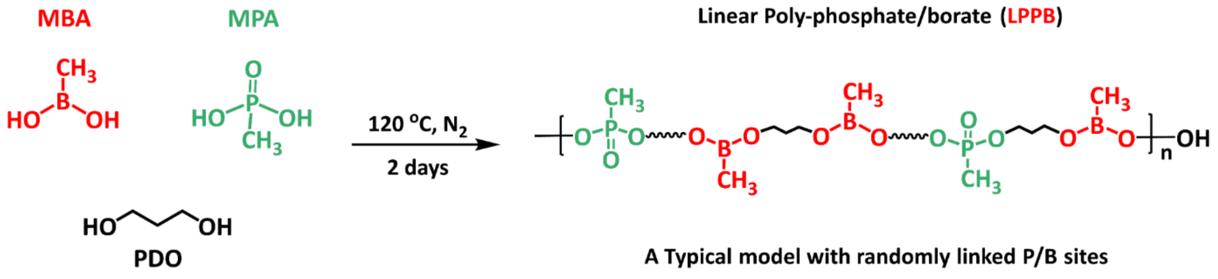

Figure S8 Synthesis route of LPPB

Figure S9 gives the IR information of the reaction monomers and the synthesized LPPB. In the spectrum of LPPB, a methylene stretching vibration appears at 2900 cm^{-1} from the esterification reaction of PDO with MBA and MPA. A broad peak at 2300 cm^{-1} corresponds to -P(CH_3)-, while the peaks at 1050 cm^{-1} belongs to the stretching vibration of B-O-C/C-O-C/P-O-C, proving the presence of BO_2/PO_2 units. Additionally, a relative decrease in the intensity

of the hydroxyl group signal in LPPB indicates the consumed hydroxyl groups, proving the proceeded reaction.

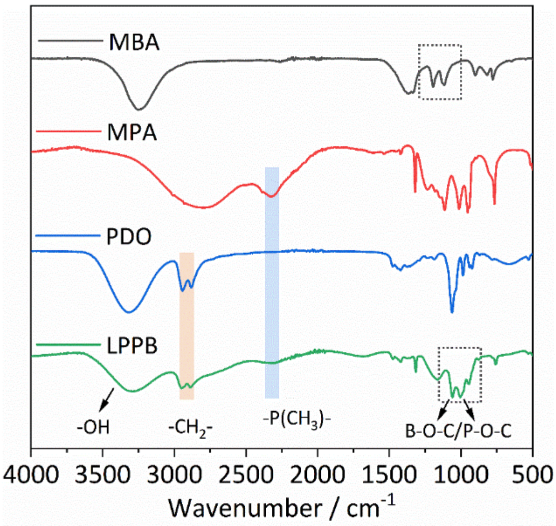

Figure S9 The FTIR spectra of the reaction monomers and the as-synthesized LPPB

S1.6. Synthesis details of HDCNs

The synthesis of HDCNs employed conventional thermosetting workflow to cast vitrimer samples. The resin formulations are outlined in **Table S11**, comprising of a hyperbranched macromonomer (HBPPB), a commercial petroleum-based epoxy resin (DGEBA, E51), an anhydride-type curing agent (MTHPA), and a small quantity of tris(dimethylaminomethyl)phenol (DMP-30, curing catalyst). Specifically, varying mass fractions of HBPPB (3 wt%, 6 wt%, 9 wt%, 12 wt%) was mixed with 80.0 g of DGEBA and stirred in a 250 ml beaker at 100 °C for 10 minutes, yielding in a yellow-transparent resin solution. The mixture was then cooled to 60 °C for the next step. Subsequently, 56.0 g of MTHPA were added and stirred at 60 ^{o}C for 15 minutes. Finally, 0.7 g of DMP-30 was dropped into the pre-polymer solution and degassed in a 60 ^{o}C vacuum for 30 minutes before casting into the mold. The resulting samples, denoted as EP-x, where x represents the mass fraction of HBPPB, were cured following a temperature procedure of 120 °C for 2 hours, 150 °C for 3 hours, and 180 °C for 2 hours. The typical reactions occurring during polymer crosslinking are described in **Figure S10**.

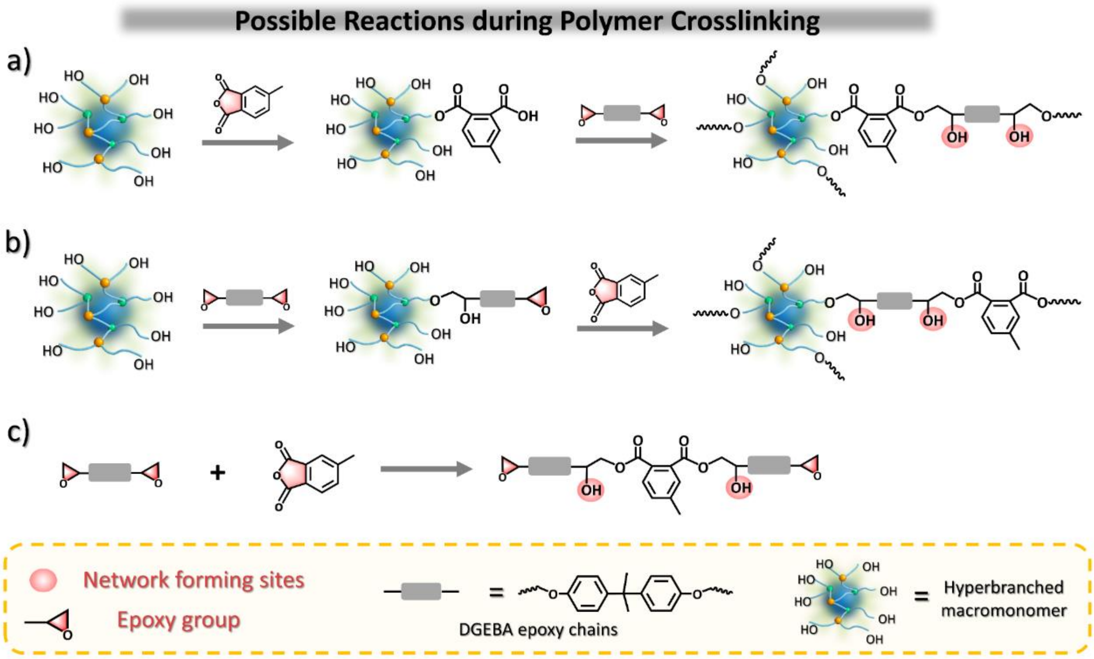

Figure S10 Typical reactions between HBPPB and epoxy/anhydride system.

In parallel, epoxy vitrimers containing different mass fractions of HBPB, HBPP and LPPB were fabricated as controls, respectively, following the same route as the HDCNs. Notably, both HBPPB and HBPB exhibited uniform resin solutions. However, HBPP shows poor

compatibility within the epoxy matrix over 6% mass content (**Figure S11a**), which can observe deposit and bulk agglomerations. This outcome proves that HBPP alone cannot serve as an effective crosslinker in such a system.

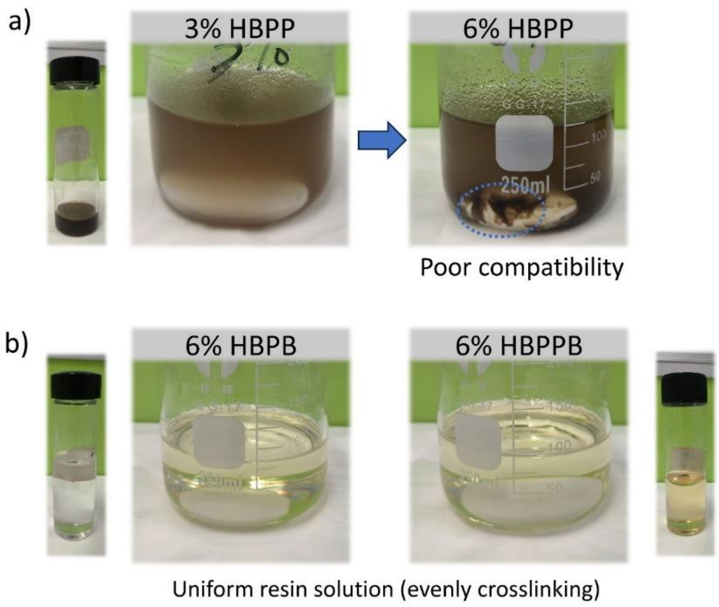

Figure S11 Compounded resin solutions showcases the poor compatibility in a) 6% HBPP/EP, and uniform solutions in both b) HBPB/EP and HBPPB/EP.

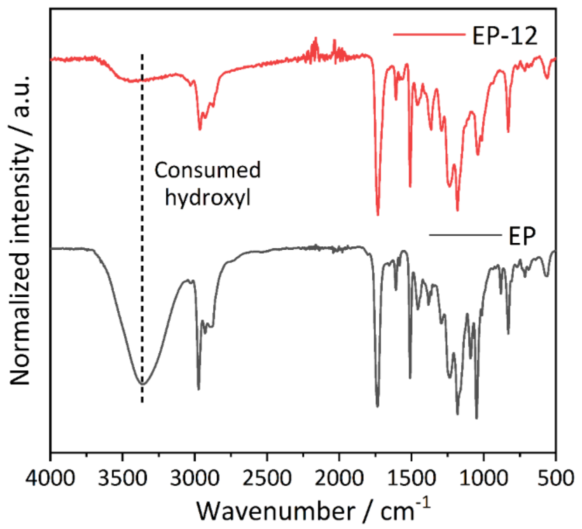

Figure S12 FT-IR spectra indicates the consumption of -OH in crosslinking of HDCNs

S1.7. Degradation, cycling case and reconfigurable experiments of HDCNs

Samples with varying concentrations of HBPPB were immersed in excess solvents (DMAc, ethanol, and deionized water) at room temperature for several days. The samples condition was recorded. Concurrently, a rapid process was initiated by elevating the system temperature up to 95°C for 6 hours. The resulting degraded product, exemplified by EP-12, was readily fabricated into fine powder through drying and grinding. For the cycling case of end-of-use vitrimer sample, 10 g of the degraded epoxy powder was thoroughly mixed with 30 g of DGEBA at 100°C for 30 minutes with stirring. Subsequently, 24 g of MTHPA and 0.24 g of DMP-30 were added following the same thermosetting procedure to fabricate the post-cycled EP for testing. The mechanical strengths of the cycled sample were assessed using an electro-mechanical tester.

In the context of reconfigurable experiments, we subjected the samples into a closed container upon heating in ethanol at 95 °C. After 6 hours, the samples were cooled to room temperature. While the thermoset EP retained its rigid form, EP-9 and EP-12 shows significant transformation from a stiff to an elastomer nature (video S1). The samples were evaluated using FT-IR, XPS detection, temperature-dependent IR, dynamic thermomechanometry, and so on.

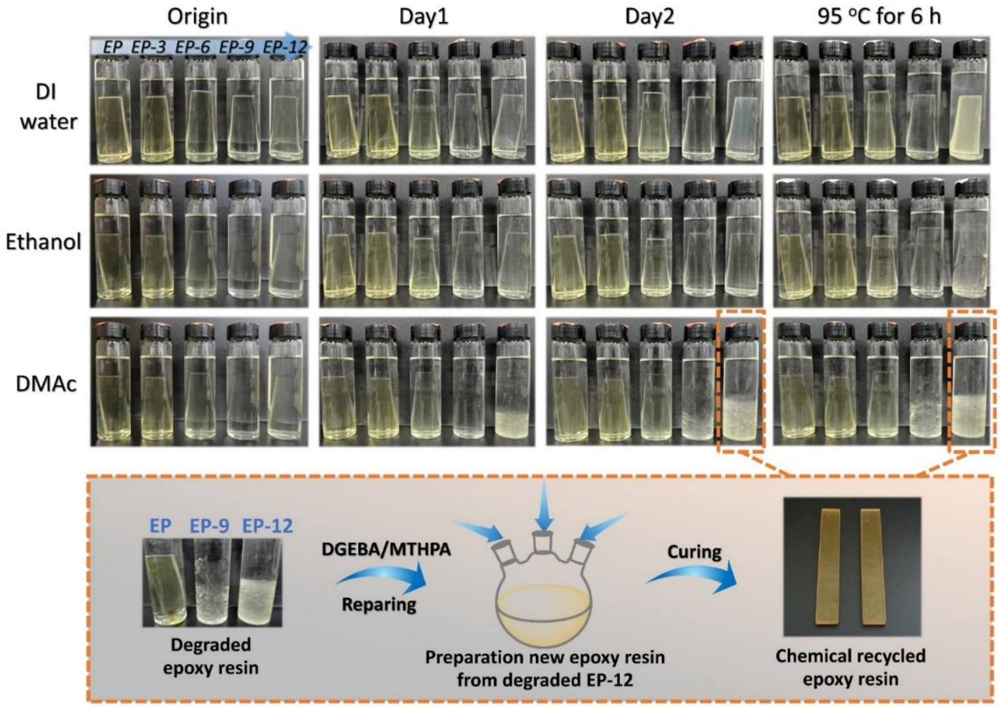

Figure S13 Photographs showing the samples with varying mass concentrations of HBPPB exposed in deionized water, ethanol, and DMAc at room temperature for several days, and at 95°C for 6 hours.

S1.8. Investigation of model reactions during vitrimer reconfiguration

a. Isolation and characterization of dissolved substance in ethanol

Prior to commencing the model reaction, the solution for immersing EP-9 was collected and subjected to rotary evaporation at 60°C for drying. As a result, a viscous substance with an approximate weight of 0.27 g was isolated from the ethanol solution, indicating a small amount of substance dissolved in the solvent. The FT-IR spectrum (**Figure S14a**) of the isolated substance exhibited infrared characteristics akin to those of MTHPA, featuring distinct C=O absorption in anhydride structure and C-O-C absorption in carboxyl ester. Further confirmation was achieved through ^{1}H-NMR analysis (**Figure S14b**), which revealed proton chemical shifts comparable to MTHPA, alongside the appearance of new signals corresponding to methyl and methylene protons in ethyl ester moieties.

These results substantiate the presence of ester derivatives of methyl tetrahydrophthalic anhydride originating from the solvent residue, supporting the occurrence of ester exchange during vitrimer reconfiguration. However, it should be noted that it is theoretically difficult to undergo complete depolymerization of polymer network, since the transesterification reaction is an equilibrium process with limited equilibrium conversion under kinetic-dominant conditions without catalysts.[6] Additionally, similar solvent-assisted dynamic reconfigurability in ethanol has also been observed under transesterification conditions.[7]

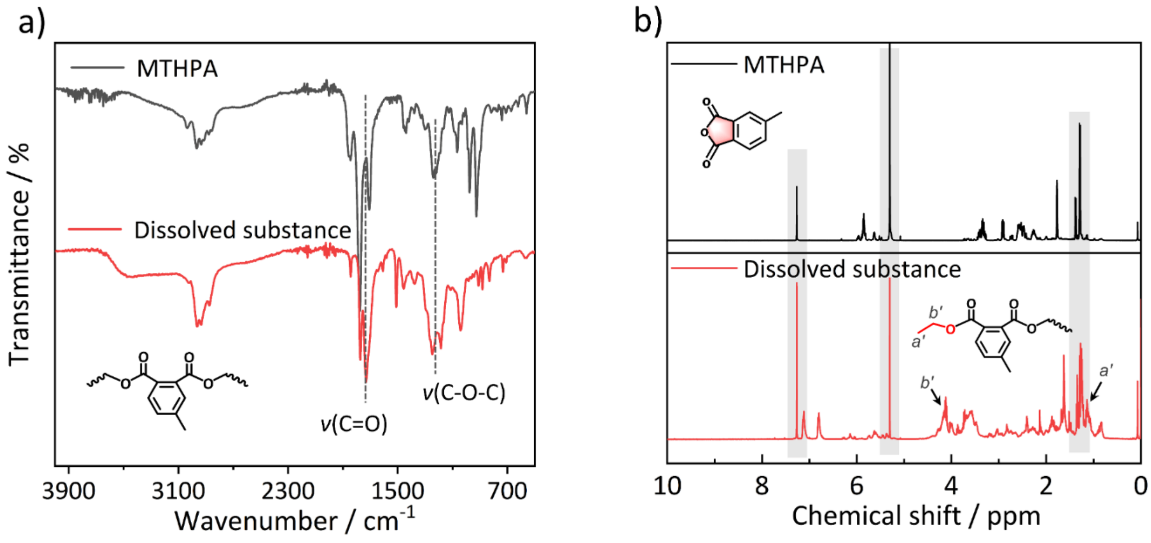

Figure S14 Characterization of the dissolved substance in ethanol after drying: a) FTIR spectrum, and b) ^{1}H-NMR compared to that of MTHPA.

To elucidate the role of ethanol as a plasticizer, the post-treated vitrimer sample was weighed after complete drying in a vacuum at 60 °C overnight. The sample weight was 6.78 g, derived from an original sample weight of 5.96 g, with 0.27 g being dissolved in the solvent. This observation indicates that the sample underwent swelling, with ethanol molecules penetrating the polymer network and potentially acting as a plasticizer to enhance chain mobility and flexibility. Indeed, the solvation effect plays a key role in this process.[8,9] Furthermore, we investigated the effects of other commonly used solvating-type plasticizers by immersing EP-9 in various solvents (**Table S3**). It was observed that reconfigurability was consistently accompanied by the swelling of sample in cases of alcohol- or ester-type solvents. However, the swelling did not necessarily lead to reconfigurability when acetone was used as the solvent. Hence, we considered that plasticizing effect might involve in reconfigurable process, but the ester exchange should be the main mechanism to drive the reconfigurable behavior of the material.

Table S3 Weight retentions of EP-9 after 95 °C heating for 6 h in varying solvating plasticizers

Solvating Plasticizers	Sample Mass (g)	Final Mass (g)	Weight Retention (%)	Reconfigurability
ethanol	5.96	6.78	113.8	Yes
methanol	5.22	5.50	105.4	Partial
ethyl acetate	5.03	5.78	115.1	Yes
acetone	5.13	5.48	106.8	No
ethanediol	5.17	5.18	100.2	No
petroleum ether	5.41	5.40	99.8	No

b. Synthesis and characterization of two model esters

The methodology of model reactions was employed for further investigation.[10] In principle, to verify the actions of hyperbranched structure, the synthesis of model compounds should involve the utilization of hyperbranched macromonomer, instead of small molecules while applicable.[11,12] Two model esters (denoted as M1 and M2) were synthesized as shown in **Figure S15** and **S17**, each featuring specific ester structures and molecular weights (**Table S4**). The synthesis subjected to the same stoichiometric ratio as the preparation of EP-9 vitrimer.

For the synthesis of M1 (Figure S16), 2 g of HBPPB was mixed with 10 g of MTHPA and heated at 120 °C for 1 hour, yielding the target pre-polymer ester M1 (M_n =18561.5 kDa, *PDI* = 1.241). The molecular weight information was provided in **Table S4**. The FT-IR evolution of synthetic M1 (**Figure S16**) revealed an increase in the absorption of C=O and C-O-C signals at 1740 cm^{-1} and 980 cm^{-1}, respectively, indicating the formation of carboxyl esters bonds.

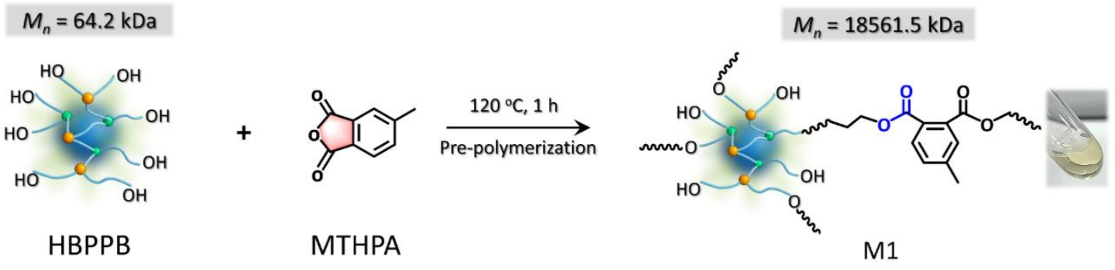

Figure S15 Synthesis of model ester M1.

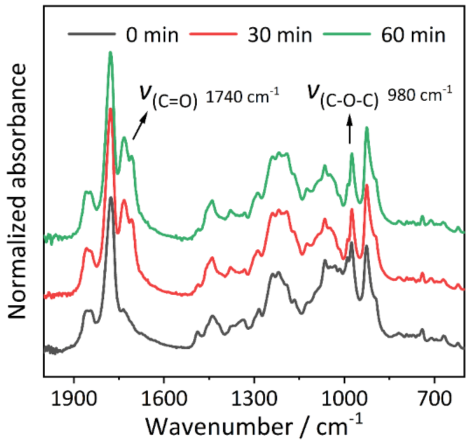

Figure S16 FT-IR evolution of the synthesis of M1 for varying reaction time

For the synthesis of M2 (Figure S17), 10 g of DGEBA was mixed with 8 g of MTHPA and stirred at 120 °C for 1 hour, resulting in target pre-polymer ester M2 (M_n = 3260.9 kDa, *PDI* = 1.462). The formation of carboxyl ester bonds was identified through FT-IR evolution (**Figure S18**), where a significant shift in the C=O signal at 1740 cm^{-1}, an increasing C-O-C absorption and a reduction in the epoxy group at 1030 cm^{-1} were observed.

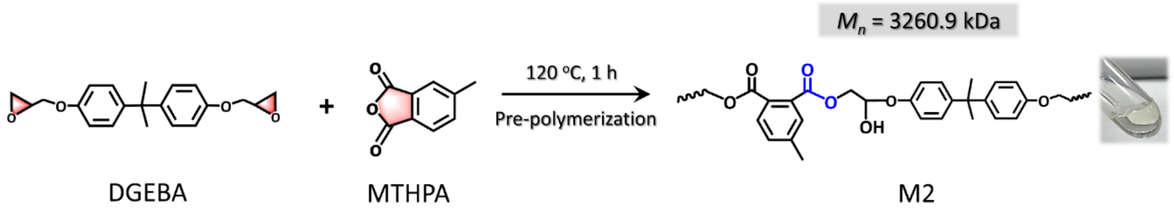

Figure S17 Synthesis of model ester M2

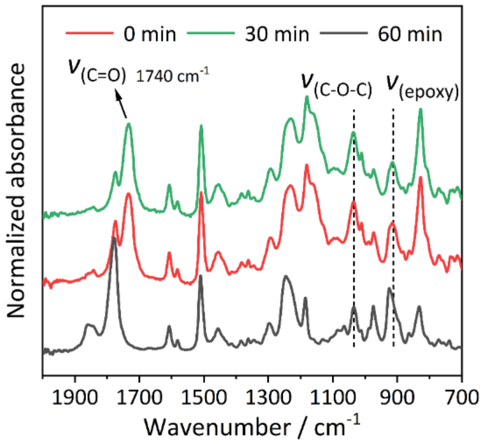

Figure S18 FT-IR evolution of the synthesis of M2 for varying reaction time

Table S4 Molecular weight information of the model esters determined by GPC-MALLs

Sample	M_n (kDa)	M_w (kDa)	M_z (kDa)	PDI (M_w/M_n)
M1	18561.5	23041.3	29259.5	1.241
M2	3260.9	4766.0	7106.9	1.462
M1-R	11189.1	14653.4	18845.2	1.31
M2-R	1427.6	1549.8	1475.0	1.086

c. Conducting model reactions

The model reactions were conducted by heating at 95 °C in ethanol. Specifically, 10 g of the as-synthesized M1 and M2 were individually mixed with 10 g of absolute ethanol. The mixtures were then placed in a flask equipped with a condenser and heated at 95 °C for varying durations of 0.5 h, 2 h, and 4 h. After the reactions, excess ethanol was removed by employing a 60°C rotary evaporator, resulting in the final viscous products (denoted as M1-R and M2-R, respectively). The molecular weights of M1-R and M2-R were both reduced as given in **Table S4**, indicating depolymerization by ester exchange between ethanol and the model esters.

Upon conducting time-dependent ^{1}H-NMR analysis, a pronounced shift in the methylene proton from 3.97 ppm (signal b, triplet) to 3.72 ppm (signal b', quartet) was observed in **Figure S19b** (right part). Besides, the signals of methyl proton (signal a') appear at 1.23 ppm with triplet peak characteristics. The normalized intensity of these signals gradually increased with reaction time, giving another evidence that the exchange reaction has taken place.

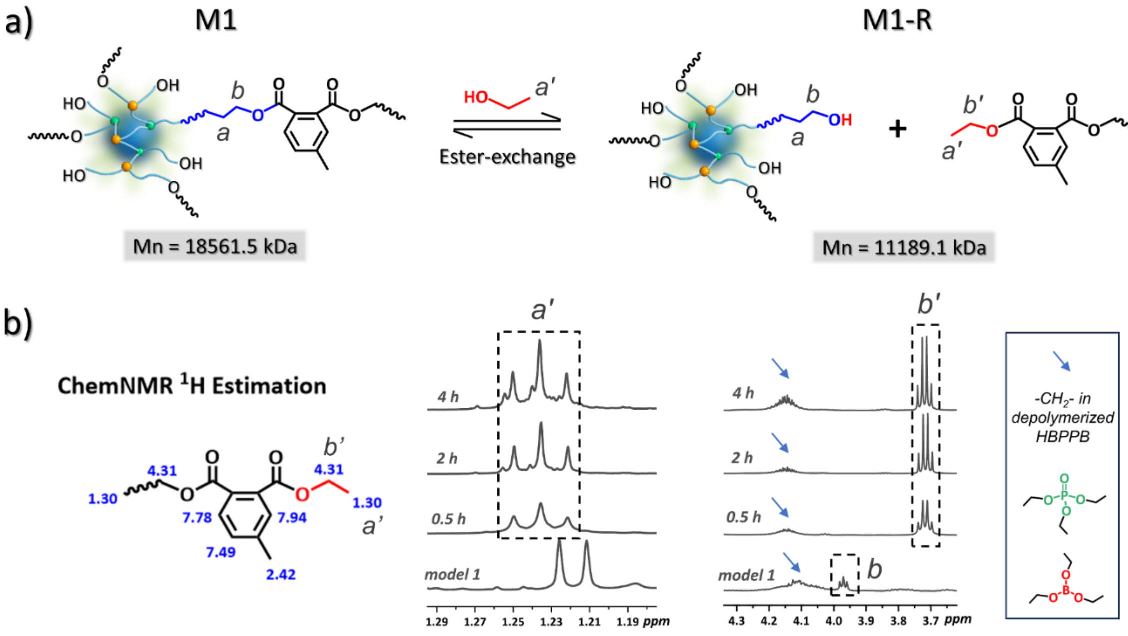

Figure S19 a) Ester exchange of molecular model 1, and b) normalized time-dependent ^{1}H-NMR of the model molecule upon 95 ^{o}C heating in ethanol.

Similarly in ester 2 (**Figure S20**), the integral area of methyl proton and methylene proton increased gradually as the reaction time. These observations indicate the occurrence of ester bond exchange between the ethanol and the model esters.

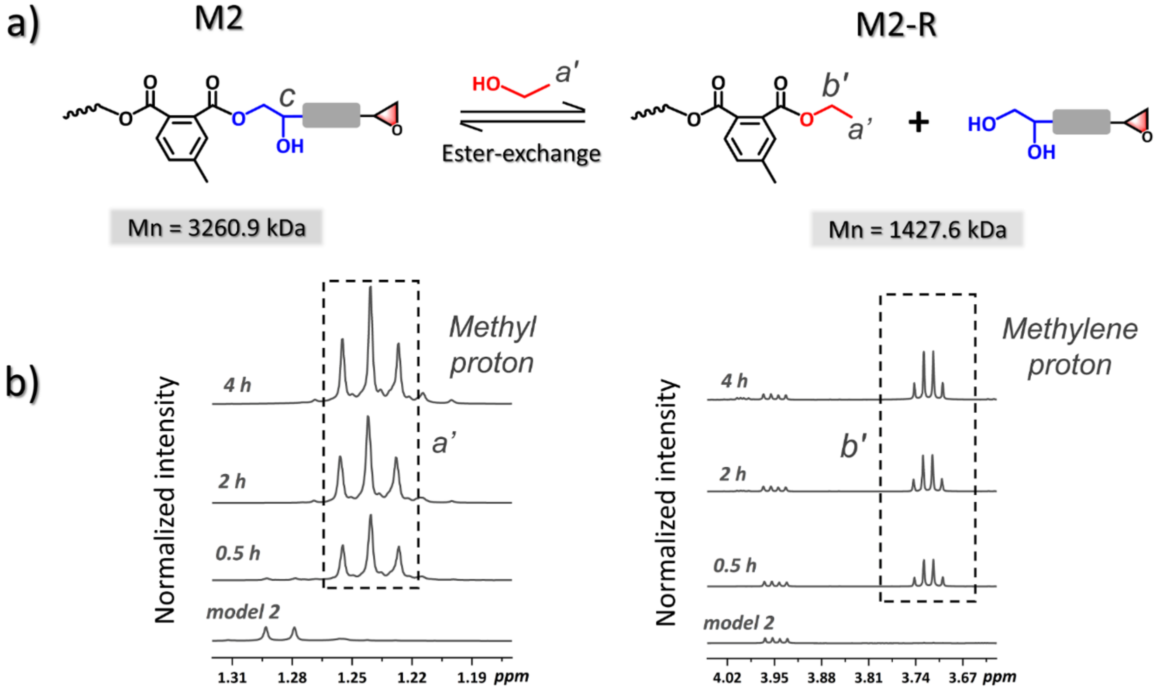

Figure S20 a) Ester exchange of molecular model 2, and b) normalized time-dependent ^{1}H-NMR of the model molecule upon 95 ^{o}C heating in ethanol.

S2. Material performance test

S2.1 The calculation of network crosslinking density

Based on the rubber elasticity theory, the crosslinking density ($d_{crosslink}$) of a polymer network can be calculated using the dynamic thermomechanometry method. The calculation is based on **Equation (2)**, with the results are listed in **Table S5.**

$$d_{crosslink} = E'/[2(1+\gamma)RT] \quad (2)$$

Where E' is the rubbery plateau storage modulus at T_g + 40 °C; γ is Poisson's ratio, generally assumed to be 0.5 when the crosslinked network is incompressible in case of a thermoset; R is the gas constant and T is absolute temperature. It should be noted that this equation is applicable for lightly crosslinked materials and therefore is used only to qualitatively compare the level of crosslinking in the casting resins.[13,14] The result for $d_{crosslink}$ is presented in the text as Figure 4c.

Table S5 DMA parameters for calculating the crosslinking density of HBPPB/EP

Samples	T_g / °C	E' at T_g + 40 °C / MPa	$d_{crosslink}$ / mol m^{-3}
EP	131.2	38.99	3.51 × 10^{-3}
EP-3	121.5	34.64	3.20 × 10^{-3}
EP-6	117.6	28.02	2.60 × 10^{-3}
EP-9	104.4	21.51	2.07 × 10^{-3}
EP-12	94.2	15.75	1.55 × 10^{-3}

[a)] T_g: glass transition temperature; E': storage modulus at three-bending mode; $d_{crosslink}$: crosslinking density of polymer

S2.2 Thermal and thermomechanical test

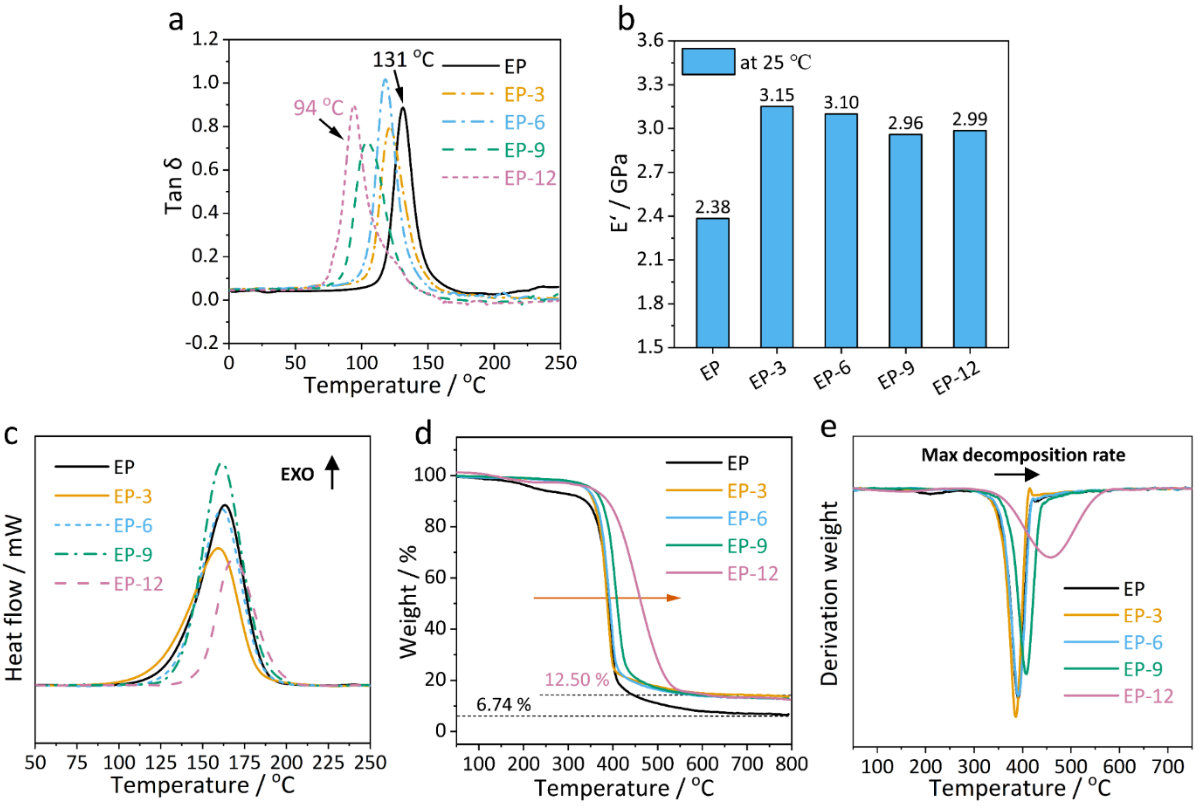

Figure S21 a) Tan δ curves, b) storage modulus (*E'*) recorded at 25 °C; c) Differential scanning calorimetry (DSC) displays similar exothermic peaks for all the pre-polymer systems; d) TGA and e) derivative thermogravimetry (DTG) curves show increase in temperature to maximum decomposition rate (T_{max}) and char yield with the incorporation of HBPPB.

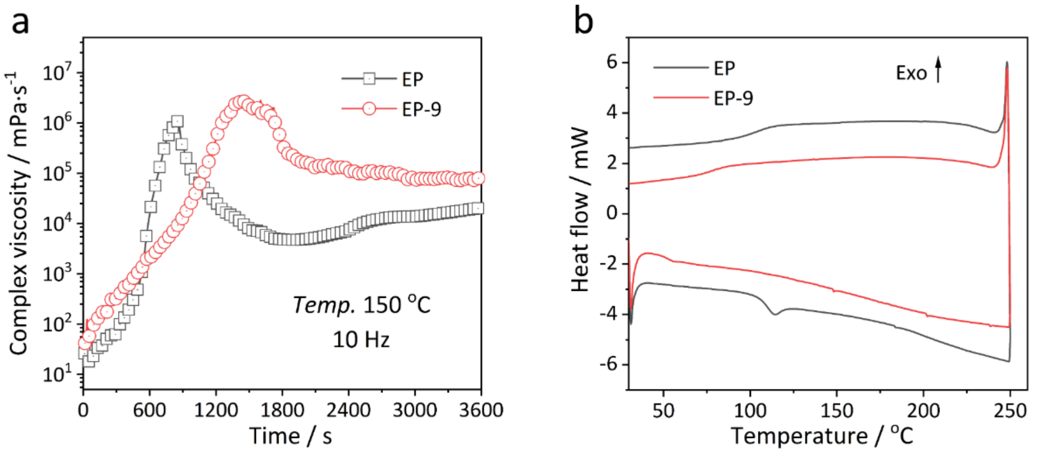

Figure S22 a) Time-sweep curves illustrating the increasing viscosity of the sample at 150 °C (The subsequent decrease is attributed to the complete curing of sample); b) Non-isothermal DSC curves indicating fully curing of EP and EP-9 without any exothermic peaks.

Table S6 Characteristic thermal and thermomechanical parameters

Samples	DSC			DMA		TGA		
	T_i (ºC)	T_{Ex} (ºC)	T_f (ºC)	E' at 25 ºC (GPa)	T_g (ºC)	$T_{5\%}$ (ºC)	T_{max} (ºC)	Char yield
EP	100	163	201	2.38	131	233	391	6.7 %
EP-3	95	159	204	3.15	121	340	387	13.8 %
EP-6	109	161	205	3.10	118	340	391	12.8 %
EP-9	121	162	207	2.96	104	352	409	12.7 %
EP-12	130	168	207	2.99	94	353	457	12.5 %

[a])T_i : Temperature at peak initial; T_{EX} : Temperature at peak value; T_f: Temperature at peak final.
[b])E': Storage modulus at three-bending mode; T_g: glass transition temperature.
[c])$T_{5\%}$: The initial decomposition temperature where 5% weight loss reaches; T_{max}: The temperature to maximum decomposition rate.

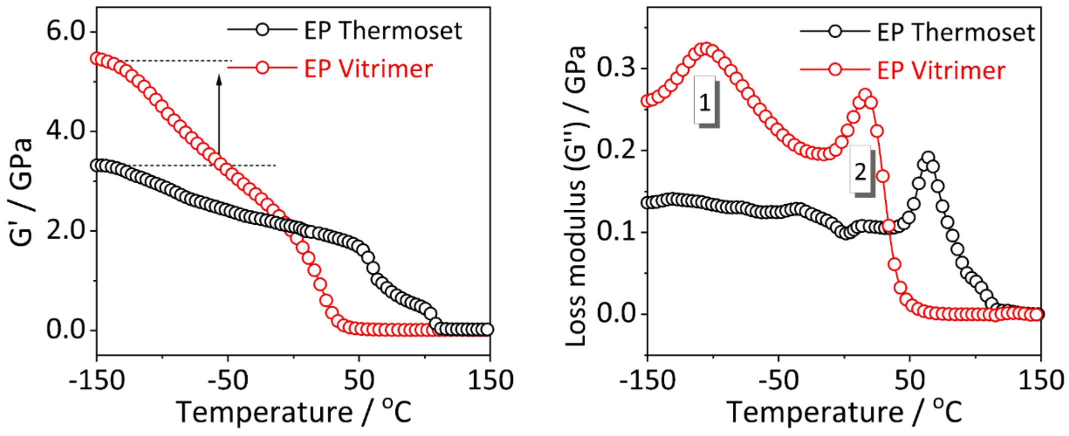

Figure S23 Dynamic thermomechanometry for EP thermoset and EP-9 vitrimer after ethanol treating: a) Storage modulus of vitrimer rapidly decreases from -150 ºC to -50 ºC, b) Loss modulus indicates a double relaxion behavior of polymer chains.

S2.3. Stress relaxation test

Stress-relaxation analyses (SRA) were performed on a Dynamic Mechanical Analyzer Q800 (TA Instrument, Waters Ltd.) with the sample dimensions of 20 mm length, 5 mm width, and 1 mm thickness under the tension mode. After reaching the testing temperature, the samples were allowed to equilibrate at the testing temperature for 10 minutes, then stretching by 5% and the relaxation of stress was monitored.

It is widely acknowledged that classical vitrimers behave linear-viscoelasticity that readily relaxes the stress above their topology-freezing transition temperature (T_v).[15] In case of a purely elastic solid, the strain (force) is instantly responsive regardless of how the stress (change) varies with time, thus we found constant curves in stress relaxation (**Figure S24**) and creep-recovery test (**Figure S26**) regarding the thermoset sample.

Nevertheless, **Figure S24b** illustrates the vitrimer partially relax to a plateau regime (approximately G/G_0 to 70%) while fails to entirely relax to the characteristic relaxation time (τ*, defined as the time required for G/G_0 = 1/e). This fact indicates that the hyperbranched dynamic crosslinking networks (HDCNs) can behave liquid-like viscoelastic properties due to the activation of dynamic bonds above T_v (describing by Arrhenius and Williams-Landel-Ferry law), but still remains their resilience and recovery ability subject to Hooke's law. Consequently, the material not only behave like a dynamic vitrimer, albeit with reduced dynamic capabilities compared to classical vitrimers, while demonstrate exceptional dimensional stability and mechanical strength even over T_g and T_v, which might attribute to permanent crosslinked sites and potentially relate to the topological crosslinking architecture.

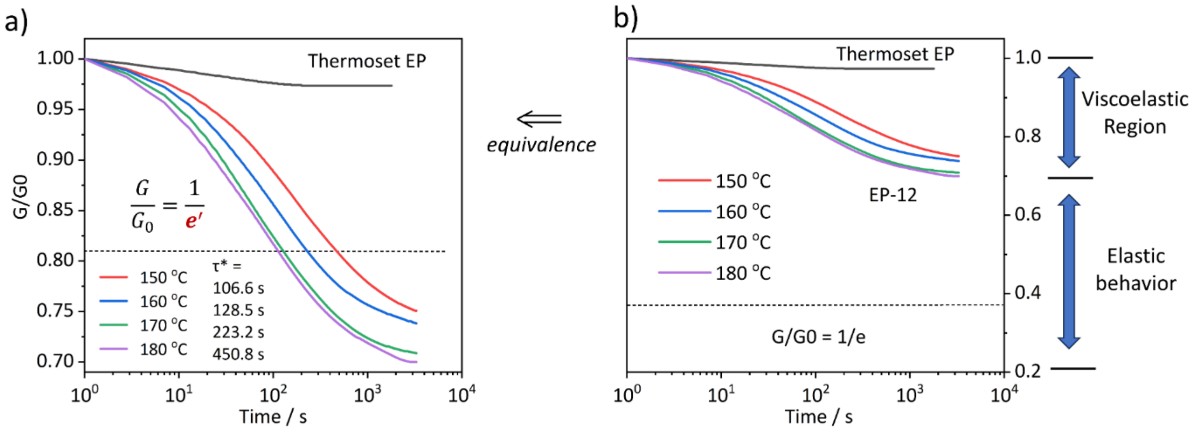

Figure S24 a) Stress-relaxation curves for an equivalence of Maxwell relaxation in viscoelastic regime to relative 1/e'. b) original stress-relaxation of EP-12 with respect to thermoset EP.

In order to determine the activation energy (E_a) and topology-freezing transition temperature (T_v), we could assign this exception as a linear-viscoelastic model combining with an ideal elastic. Considering the complexity and limitations associated with solving these non-linear viscoelastic issues,[16,17] we simplify the stress-relaxation curves by dividing them at the boundary of the stress-relaxation plateau (Figure S24b to S24a). The upper region was assigned as viscoelastic relaxation, while the region below the plateau is approximately denoted as an ideal elastic polymer. Based on the equivalence, we can assume the sample fully undergoes its viscoelastic relaxation once they reach the plateau. The characteristic relaxation time (τ^*) can be therefore determined using a modified Maxwell equation until G/G_0 relax to $1/e'$ (**Equation 3**),[18,19] where e' is modified by a correction factor k in **Equation 4**.

$$G = G_0 e'^{-\frac{t}{\tau^*}} \tag{3}$$

$$\frac{1}{e'} = \frac{1}{e}(1-k) + k \tag{4}$$

Where k is a correction factor (value at which G/G_0 reaches to the relaxation platform), assuming to 0.7 in this system. The activation energy (E_a) could be determined from the slope in an Arrhenius-plot (**Figure S25a**) using **Equation 5-6** as follows.

$$\tau^* = \tau_0 e^{\frac{E_a}{RT}} \tag{5}$$

$$\ln(\tau^*) = \ln(\tau_0) + \frac{E_a}{RT} \tag{6}$$

The topology-freezing transition temperature (liquid-to-solid transition temperature, T_v) is defined as the point at which a vitrimer exhibits a viscosity (η) of 10^{12} Pa s, which may therefore be determined using Maxwell's relation (**Equation 7**) and E' determined from DMA.[20] τ^* was determined as ca. 1.9×10^5 at T_v. The Arrhenius relationship was then extrapolated to determine T_v as shown in **Figure S25b**.

$$\eta = \frac{1}{3} E' * \tau^* \tag{7}$$

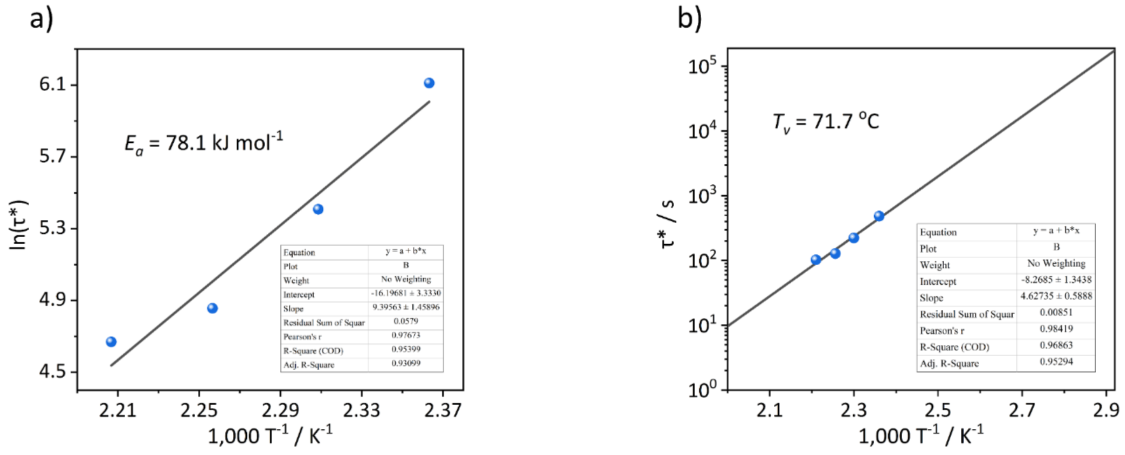

Figure S25 Arrhenius analysis of a) ln(τ*) versus 1000/T and b) τ* versus 1000/T to determine E_a and T_v.

S2.4. Creep-recovery test

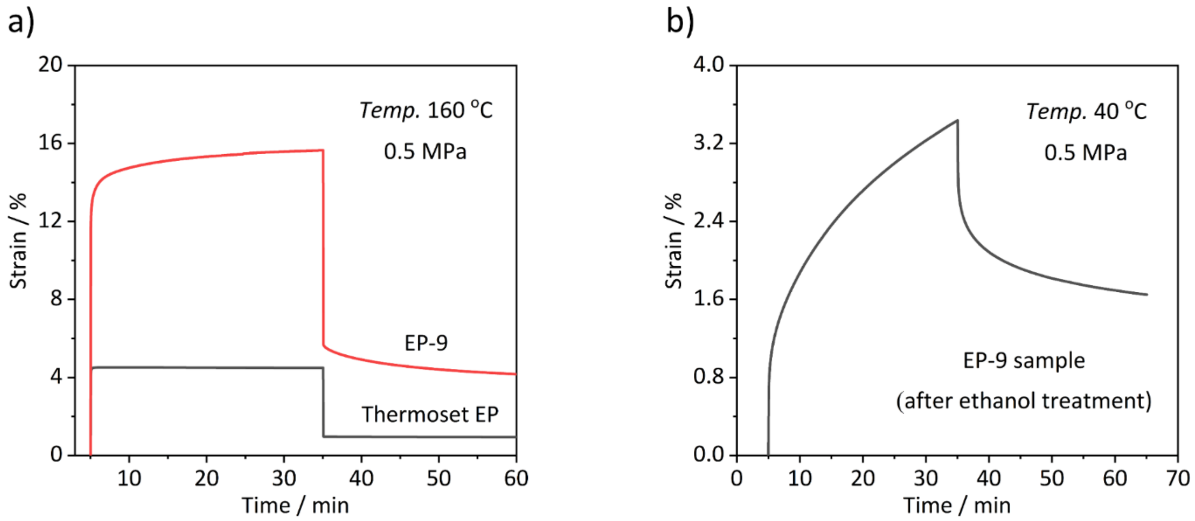

Figure S26 Tensile creep-recovery behavior of a) EP-9 in comparison with thermoset EP; b) EP-9 sample after treating with ethanol shows creep-deformation characteristic at 40 ^{o}C.

S2.5 Comparison of degradation and reconfigurable properties

In cases of three similar structures (LPPB, HBPP, and HBPB), their degradation and reconfigurable performance were evaluated and compared with that of the hyperbranched crosslinker (HBPPB).

a. Degradation and reconfigurable properties of LPPB/EP

For the degradation (**Figure S27**), LPPB/EP also underwent swelling and fragmentation when exposed to DMAc, while the weight retention of 12LPPB/EP is higher than that of 12HBPPB/EP, and its *E'* and T_g value is significantly lower than the thermoset epoxy and 12HBPPB/EP.

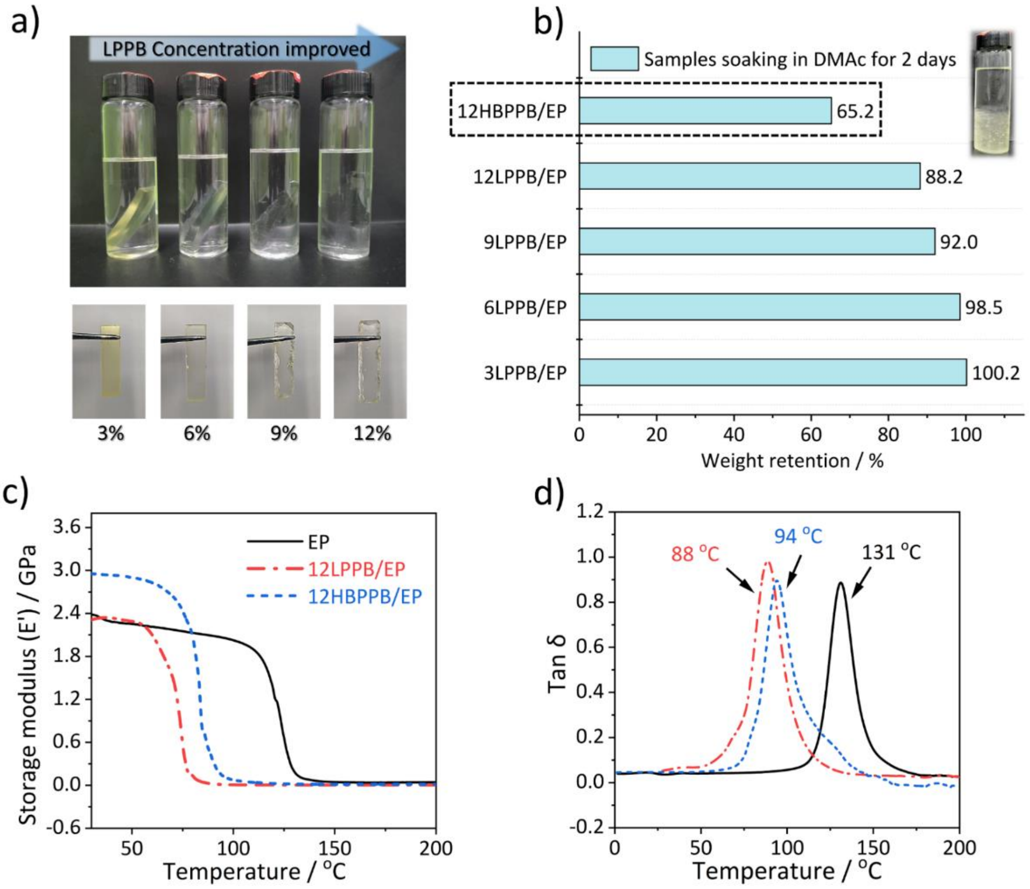

Figure S27 Photographs of a) the samples soaked in DMAc at ambient condition for 2 days, b) weight retentions as compared to 12HBPPB/EP; Dynamic thermomechanical analysis for c) storage modulus and d) glass transition temperature.

For the reconfigurable performance (**Figure S28**), notably, the ethanol-induced dynamic network reconfiguration properties were not well reflected in LPPB/EP. Not only was its storage modulus inferior to 9HBPPB/EP sample at low temperature (-100 °C, Figure S27b), but

also the network reconstruction has not been well recognized, as its tan δ curves shows two distinct peaks (Figure S28c). For the judgement, the same amount of BO_3/PO_3 and OH groups are linearly introduced, the degradation and reconfigurable properties is not good as the hyperbranched crosslinked one.

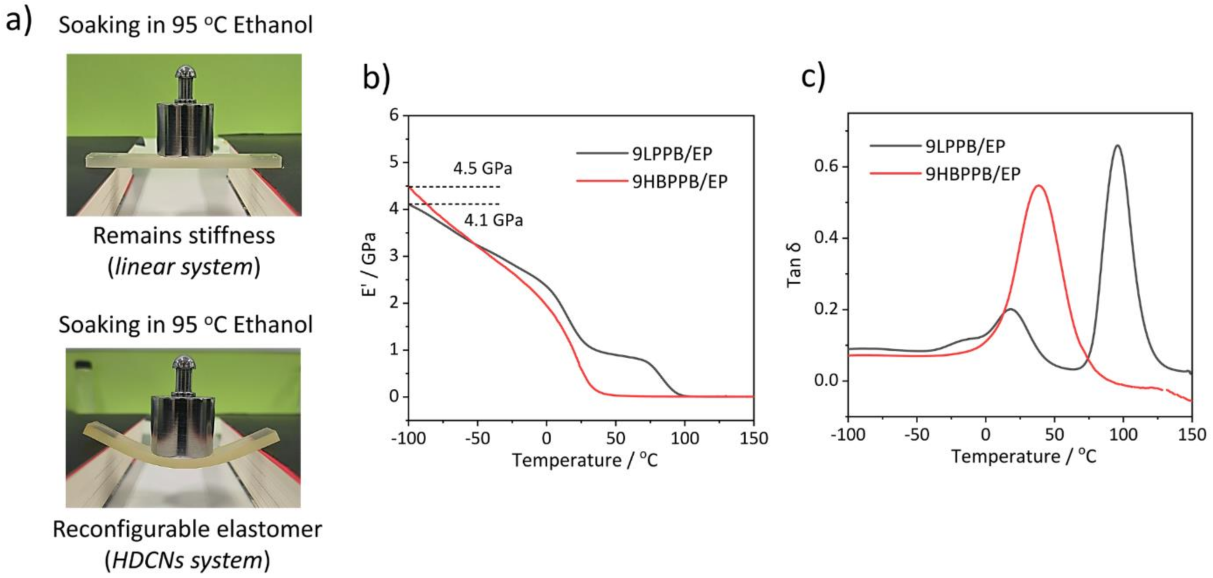

Figure S28 Reconfigurable performance of the linear system as compared to the HDCNs: a) photographs showing the samples after heating in 95 °C ethanol for 6 h in a closed container; b) storage modulus and c) tan δ curves.

a. Degradation performance of HBPP/EP and HBPB/EP

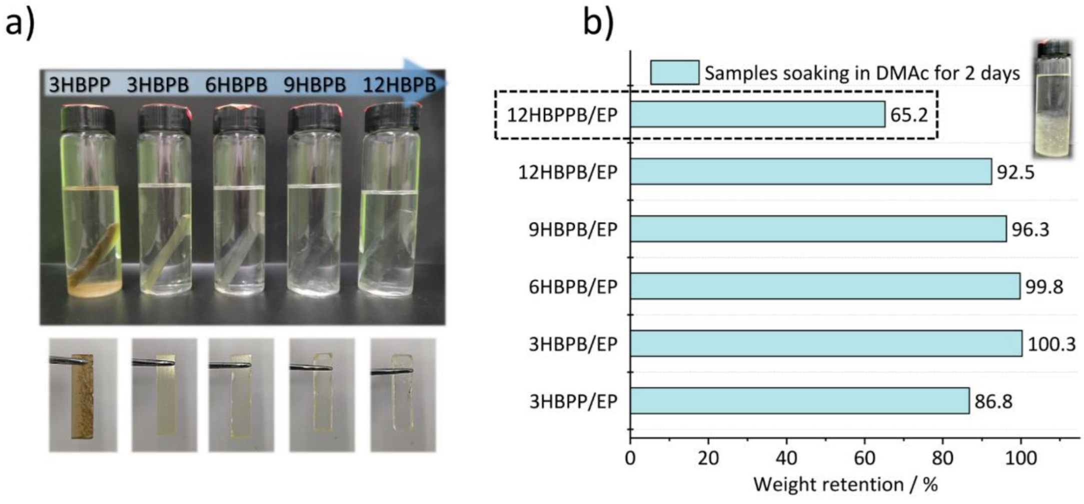

Figure S29 Photographs of a) the samples soaked in DMAc at ambient condition for 2 days, b) weight retentions as compared to 12HBPPB/EP.

S2.6. Mechanical performance test

S3.6.1. Mechanical properties of HBPPB and its blend of HBPPB

Table S7 Flexural , tensile and impact strength of EP and its blends with HBPPB

Run	Flexrual strength / MPa	Impact strength / kJ mol^{-1}	Tensile strength / MPa	Impact abosrbing energy / J
EP	106.1 ± 3.5	12.9 ± 1.4	52.1 ± 6.3	0.52 ± 0.06
EP-3	132.0 ± 0.4	25.7 ± 2.0	81.2 ± 8.6	1.06 ± 0.15
EP-6	126.0 ± 1.2	16.5 ± 1.8	77.9 ± 8.5	0.66 ± 0.06
EP-9	129.5 ± 0.3	15.7 ± 1.7	79.6 ± 4.2	0.70 ± 0.09
EP-12	121.3 ± 3.9	15.4 ± 1.2	66.0 ± 2.8	0.70 ± 0.07

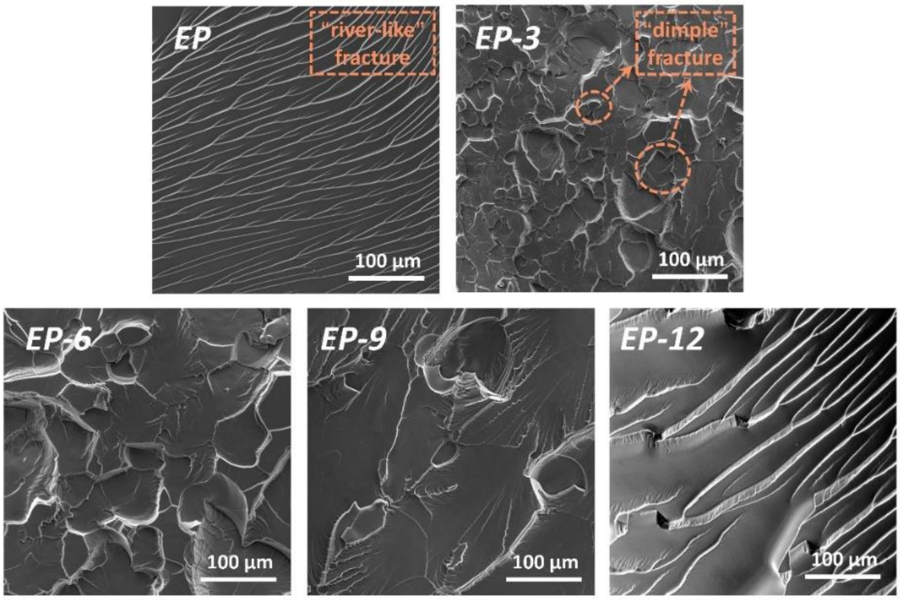

Figure S30 Impact fracture surface showing a brittle feature (rive-like) transformed to tough feature (dimple-like fracture) with increasing content of HBPPB.

a. Flexural stress-strain curves and its calculations

The flexural test determines the flexural stiffness and strength properties of materials, indicating small deformation for the stiffness thermoset feature at room temperature. The test adapted an arbitration speed of 2 mm min^{-1} with a sample dimension of 80 mm length, 15 mm

width, and 4 mm thickness. According to ASTM D7264 standard, the flexural stress (σ) can be calculated for any point on the load-deflection curves by the following equation:

$$\sigma = \frac{3PL}{4bh^3} \tag{8}$$

Where:

σ = maximum strain at the outer surface, mm/mm [in./in.],

P = applied force, N [lbf],

L = support span, mm [in./in.],

b = wide of beam, mm [in./in.], and

h = thickness of beam, mm [in./in.].

The flexural strain (ε) at the outer surface of materials occurs at mid-span is calculated as follows:

$$\varepsilon = \frac{6\delta h}{L^2} \tag{9}$$

where:

ε = maximum strain at the outer surface, mm/mm [in./in.],

δ = mid-span deflection, mm [in./in.],

L = support span, mm [in./in.] and

h = thickness of beam, mm [in./in.].

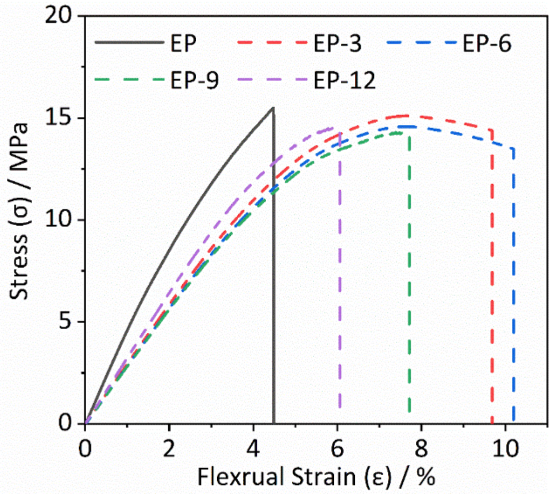

Figure S31 Typical flexural stress-strain curves of EP and its blends with HBPPB.

b. Tensile test curves

Dumbbell-shaped samples were prepared for the tensile test of HBPPB/EP according to ASTM D638-14, with an effective gauge dimension of 20 mm length, 4.5 mm width, and 2 mm thickness. The samples were stretched at the arbitration speed of 2 mm min^{-1}. Test at least five specimens for each sample in the case of isotropic materials.

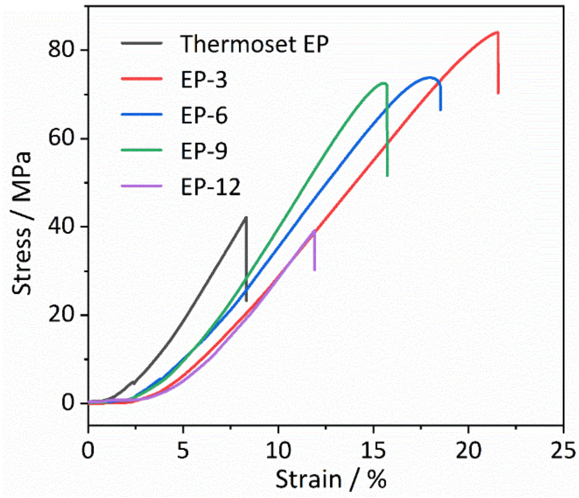

Figure S32 Typical tensile stress-strain curves EP and its blends with HBPPB.

S3.6.2. Mechanical properties of EP and its blend with LPPB, HBPP and HBPB.

The mechanical properties of EP and its blends with LPPB, HBPP and HBPB was tested to compare their strengthening and toughening effect with that of HBPPB. Test at least five specimens for each sample in the case of isotropic materials. The typical flexural stress-strain curves of LPPB/EP, HBPP/EP and HBPP/EP are given in **Figure S33-34**, with the test results summarized in **Table S8**.

Table S8 Flexural and impact strength of LPPB/EP, HBPP/EP and HBPB/EP

Run	Flexrual strength / MPa	Impact strength / kJ mol^{-1}
EP	106.1 ± 3.5	12.9 ± 1.4
3LPPB/EP	122.7 ± 4.8	17.1 ± 3.2
6LPPB/EP	130.6 ± 3.4	24.6 ± 5.8
9LPPB/EP	111.9 ± 1.4	17.9 ± 1.6
12LPPB/EP	97.4 ± 2.9	9.1 ± 2.9

3HBPP/EP	108.6 ± 4.8	11.6 ± 3.2
3HBPB/EP	111.3 ± 6.1	16.7 ± 4.9
6HBPB/EP	129.4 ± 4.1	17.3 ± 5.6
9HBPB/EP	108.2 ± 4.8	14.9 ± 0.8
12HBPB/EP	98.6 ± 3.2	14.8 ± 2.7

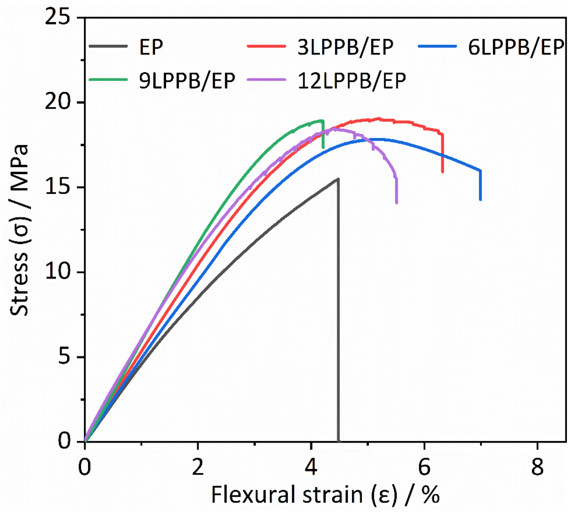

Figure S33 Typical flexural stress-strain curves of EP and its blends with LPPB.

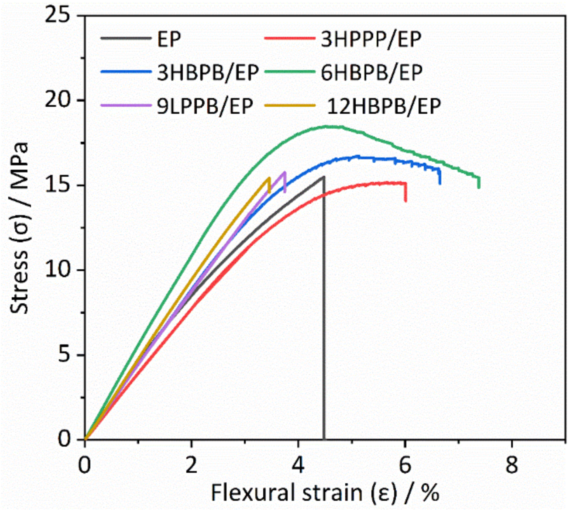

Figure S34 Typical flexural stress-strain curves of EP and its blends with HBPP and HBPB.

S3.6.3. Tensile and flexural properties of carbon fiber-reinforced polymers (CFRPs)

a. Tensile test for CFRPs

We fabricate the CFRPs to demonstrate a cycling case of CFs from the resin matrix, and facilitate a comprehensive mechanical comparison with the native epoxy composite. The CFRPs were composed of approximately 45% mass fraction of carbon fiber (CFs, T300, K12) by handling lay-up, and then thermal processing in a vacuum. The tensile test specimens adapted a gauge sample dimension of 250 mm length, 25 mm width, and 2 mm thickness, stretching at the speed of 2 mm min^{-1} (ASTM D3039 standard). Test at least five specimens for each sample. Obviously, the fabricated CFRPs demonstrated superior stretchability and toughness compared to the native epoxy composite (**Figure S35-36**), underscoring the enhanced performance of CFRPs even with the limitations in fiber content (over 90% in practical application).

Table S9 Tensile and flexural test of CFRP and its blend with HBPPB

Run	Tensile strength / MPa	Flexural strength / MPa	Elongation at break / %
EP/CF	81.2 ± 6.8	158.2 ± 3.8	9.0
3EP/CF	100.0 ± 8.1	195.6 ± 3.3	11.1
6EP/CF	91.4 ± 4.3	239.7 ± 12.7	8.2
9EP/CF	115.3 ± 3.7	228.7 ± 5.4	17.7
12EP/CF	127.6 ± 9.9	225.5 ± 4.3	16.7

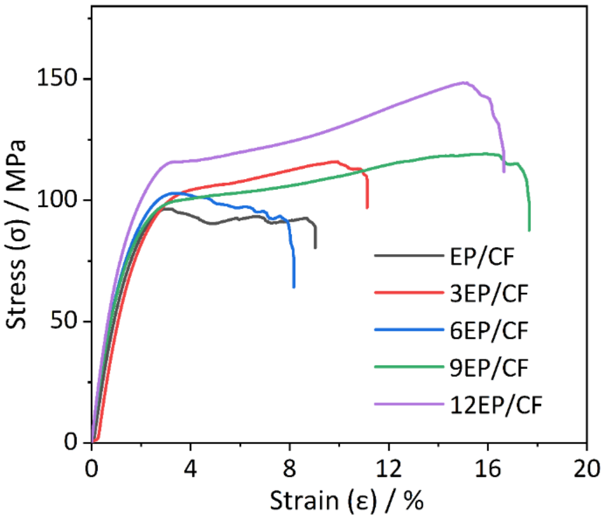

Figure S35 Typical stress-strain curves for the tensile test of CFRPs and its blend with HBPPB.

b. Flexural test for CFRPs

The flexural test was performed on a three-point flexural beam mode according to ASTM D7264-2015, with the sample dimension of 80 mm length, 12.5 mm width, and 2 mm thickness, following the stretching speed of 2 mm min^{-1}. Test at least five specimens for each sample. The strain-stress is calculated using Equation 7-8. The composite with crosslinking HBPPB shows superior flexural strength than the native epoxy composite.

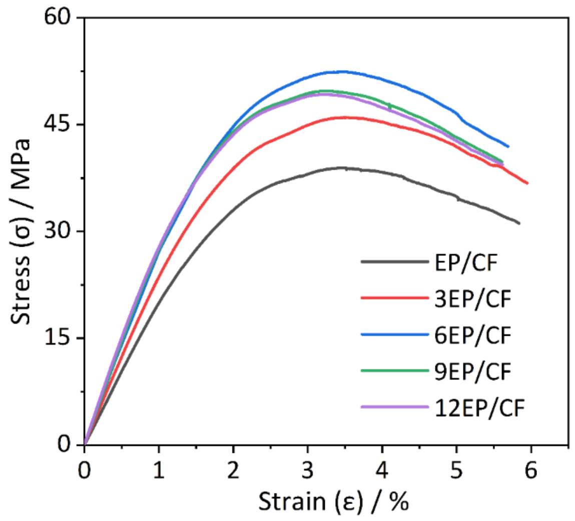

Figure S36 Typical flexural force-displacement curves for CFRPs and its blend with HBPPB.

S2.7. Solvent-resistance test

Given the significance of solvent resistance for epoxy resins in practical applications, the solvent-resistant test was performed by soaking samples of approximately 0.8 g in 10 mL solvents (including 0.1 M HCl solution, 0.1 M NaOH solution, N, N-dimethylformamide (DMF), N, N-dimethylacetamide (DMAc), N-methyl-2-pyrrolidone (NMP), dimethyl sulfoxide (DMSO), dichloromethane (DCM), toluene (TOL), acetonitrile (ACN), ethyl acetate (EtOAc), ethanol (EtOH), methanol (MeOH), and petroleum ether (PE)) at the ambient condition for 2 days.

Then, the insoluble sample was dried at 80 °C vacuum for 24 hours to a constant weight. The weight retention (%) was determined using the final sample weight dividing the original weight. The results are shown as follows.

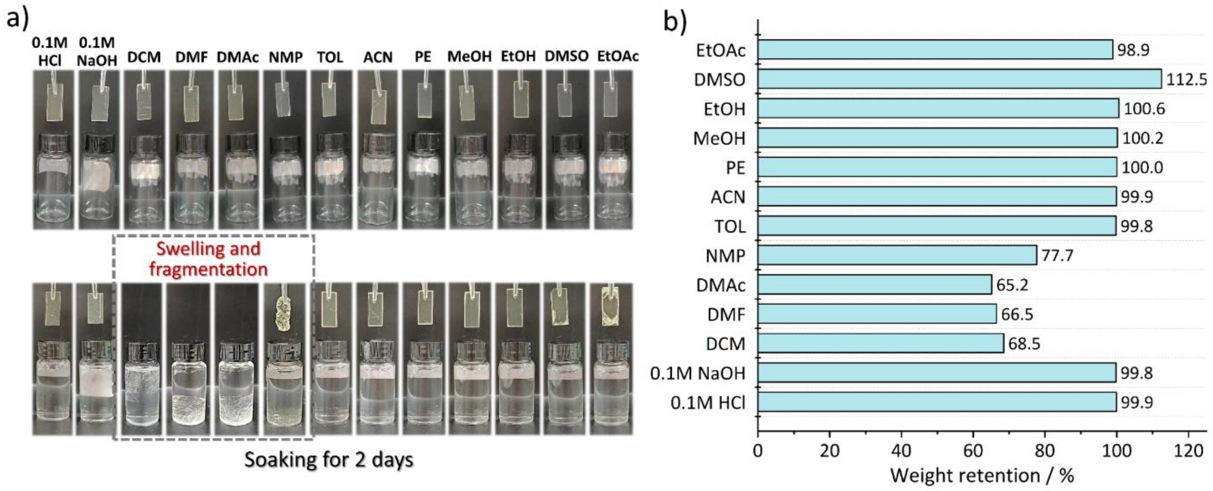

Figure S37 a) Photographs of the EP-12 samples exposed to 0.1 M HCl, 0.1 M NaOH and different organic solvents at the ambient condition for 2 days, b) weight retention of the final sample being soaked and then dried.

S2.8. XPS surveys of degradation and reconfigurable vitrimer

In conjunction with the FTIR results, X-ray photoelectron spectroscopy (XPS) was employed to assess the bonding condition following sample degradation.[21] The sample was fully dried and ground into powder for detection. Informed by XPS spectrum (C1s is corrected to 284.8 eV for each spectrum), the beta-hydroxy ester was partially broken after soaking in DMAc, as evidenced by its heightened and broader O-H/COOH intensity in O1s spectra, as well as the relatively lower signals of C=O and C-O-C in C1s spectra. Additionally, high-resolution XPS revealed very weak signals of B and P in the degraded sample, suggesting the depolymerization of HBPPB.

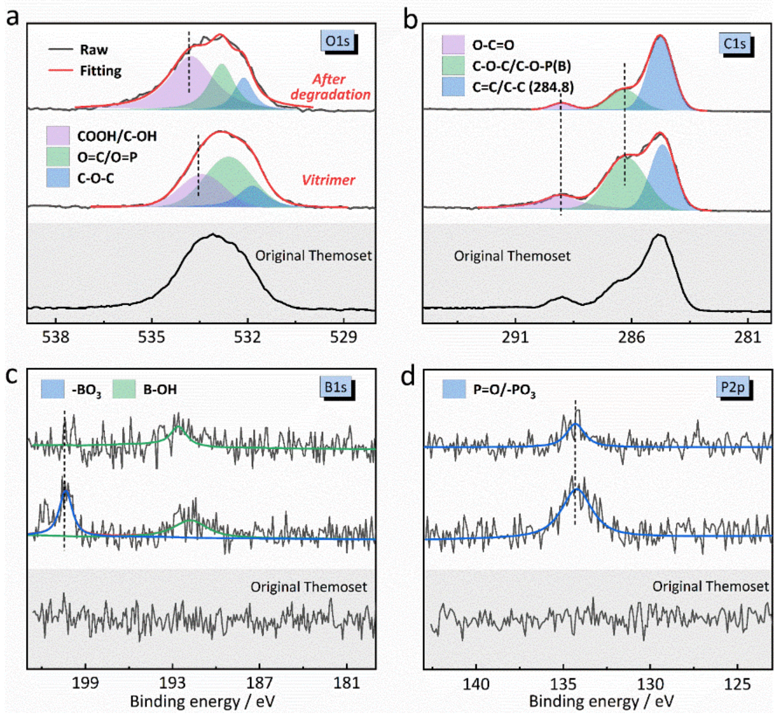

Figure S38 XPS surveys of EP-12 sample after degradation in DMA: high-resolution XPS for a) O1s, b) C1s, c) B1s and d) P2p.

To validate the reconfigurable nature of samples, the dynamic ester exchange was further confirmed using XPS analysis. The high-resolution O1s spectrum exhibited a notable shift following after the samples heating in ethanol, which is attributed to the ester exchange between ethanol and the ester moieties for resulting network reconstruction.

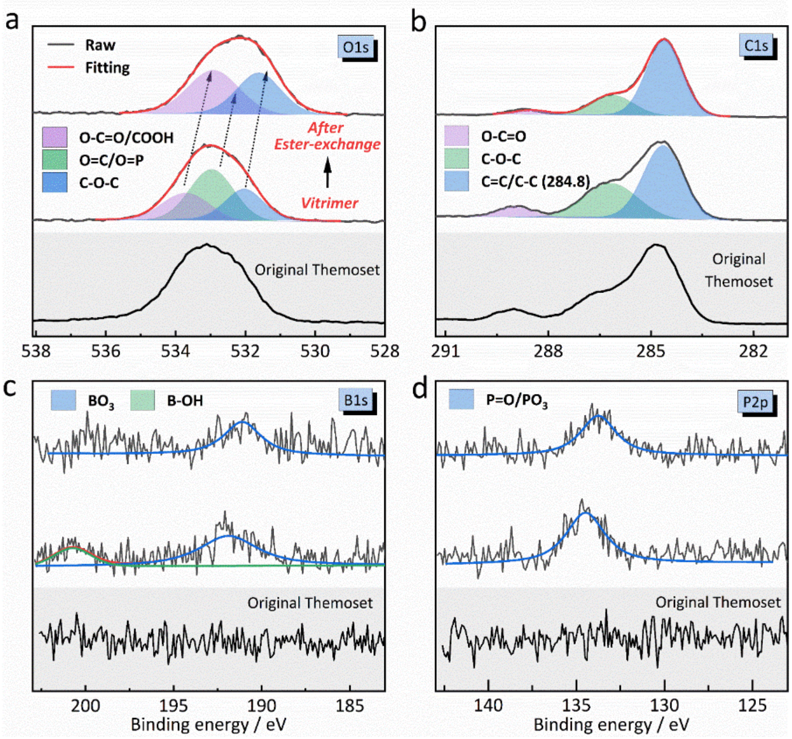

Figure S39 XPS surveys of EP-9 sample after heating in ethanol: high-resolution XPS for a) O1s, b) C1s, c) B1s and d) P2p.

S2.9. Transmittance performance test

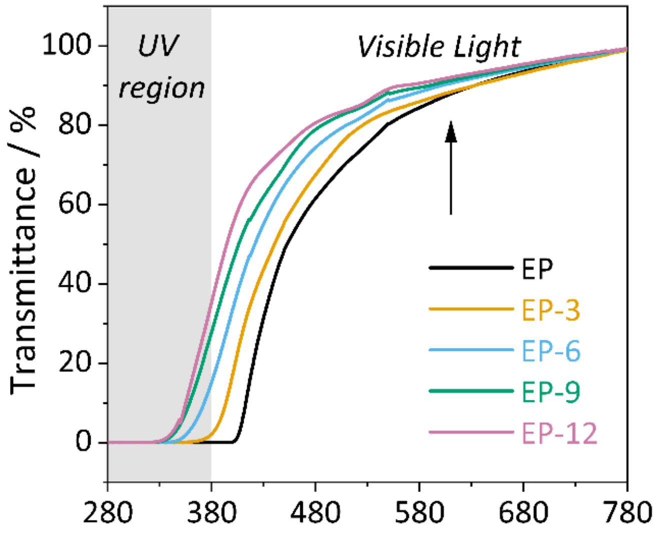

Figure S40 UV-vis transmission spectra of EP and its blends with HBPPB.

The transparency of materials was measured using a UV-vis spectrophotometer. The average transmittance over the visible light (220-380 nm) and UV light (380-780 nm) was obtained by integrating the measured data, as given in the main text in Figure 4h.

S2.10. Fire-retardancy test and mechanism investigation

The combination of phosphorus and boron exhibits remarkable efficacy as a fire-retardant strategy for polymeric materials.[22,23] Consequently, the evaluation of material's thermal hazards, smoke release, and toxic volatiles was conducted through cone calorimeter experiments. The corresponding parameters are listed in **Table S10**. Totally, the incorporation of HBPPB reduces total heat release (THR), peak heat release rate (pHRR), total smoke production (TSP), smoke production rate (SPR) and CO&CO_2 production (**Figure S41a-f**). Notably, when compared to neat EP, EP-9 exhibits a 20.7 MJ/m^2 decrease in THR, indicating improved fire resistance. Particularly, the TSP value significantly decreased from 50.6 m^2 to 33.7 m^2, implying reduced smoke suffocation during fire incidents. Besides, a fire hysteresis before 350 s was recognized, as evidenced by the delayed time to peak heat release rate (pHRR) and the peak smoke production rate (pSPR) in EP-9 compared to the thermoset EP. In a real combustion scenario, the burning process can be categorized into two stages: the main burning zone and the afterburning zone. The main burning region (100 ~ 300 s) revealed a decrease in pHRR from 764.8 to 581.1 kW m^2, while the afterburning stage nearly disappears, indicating the better fire retardancy of vitrimer.

Table S10 Some characteristic data from CCT test.

Samples	EP	EP-9	EP-12
TTI (s)	108	121	120
pHRR (kW/m^2)	764.8	619.1	581.1
FGR	3.64	2.58	2.29
FPI	0.14	0.20	0.21
THR (MJ/m^2)	124.8	113.3	104.1
TSP (m^2)	50.6	40.6	33.7
mMLR (g/s)	0.089	0.092	0.077
av-EHC (MJ/kg)	22.8	23.8	24.5
COY/CO_2Y(%)	4.05	5.18	5.77
Char yield (%)	4.36	12.31	12.94

[a]*FGR*: fire growth rate (*pHRR* / time to *pHRR*); *FPI*: fire performance index (*TTI* / *pHRR*)
[b]*TTI*: time to ignition; *mMLR*: mean mass loss rate; *av-EHC*: average effective heat combustion; *COY*: yield of carbon monoxide; *CO_2Y*: yield of carbon dioxide.

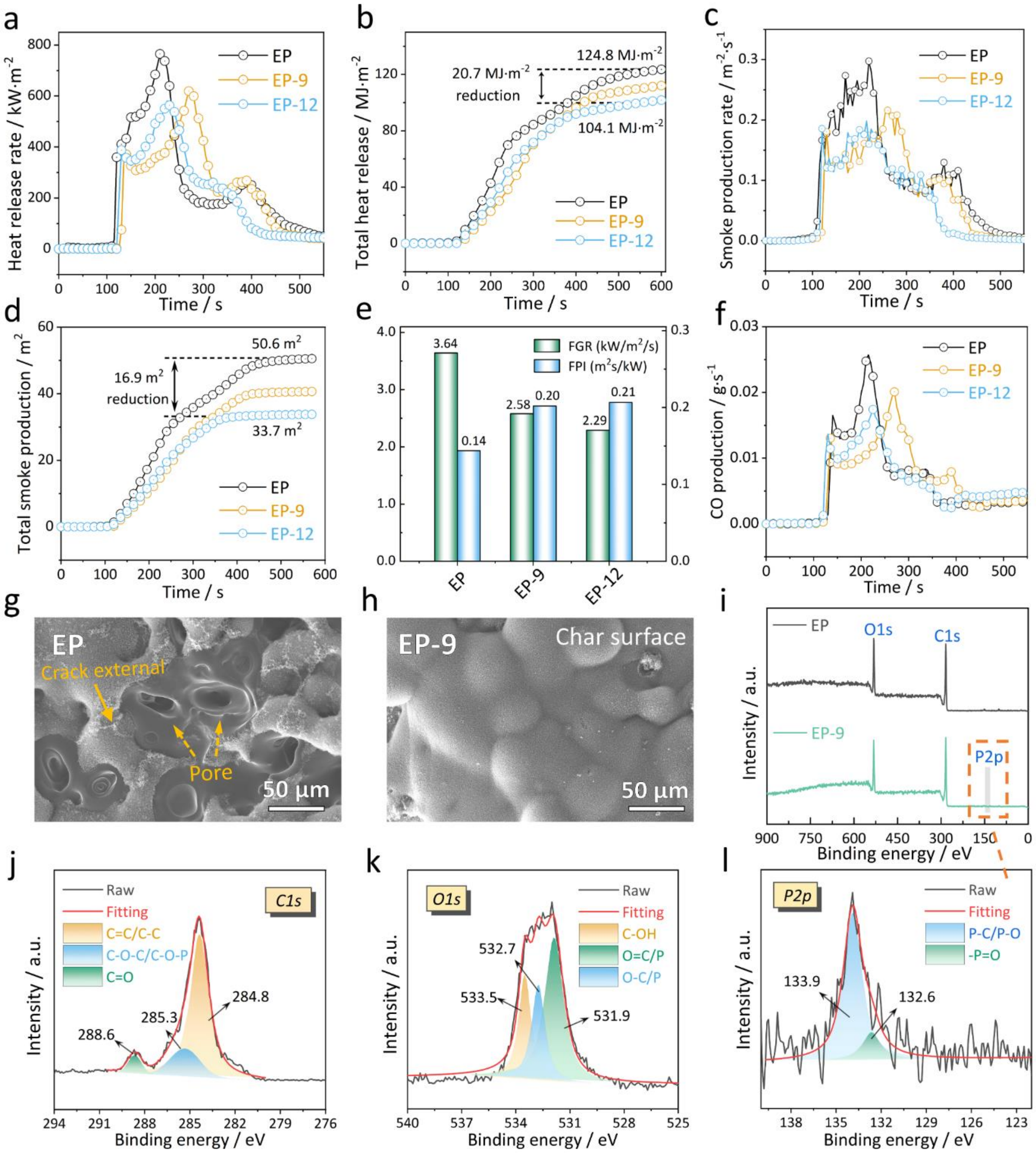

Figure S41 Cone calorimeter test records: a) heat release rate; b) total heat release; c) smoke production release; d) total smoke production; e) fire growth rate and fire performance index; f) CO production. SEM morphology of char surface: g) neat EP and h) EP-9. XPS surveys of char residue of EP-9: i) full spectrum and high-resolution spectrum of j) C1s, k) O1s, l) P2p.

Notably, the reduction in THR from 124.8 kJ m^2 to 104.1 kJ m^2 was accompanied by an increase in average effective heat combustion (av-EHC, heat release per unit), attributed to the early decomposition of HBPPB, which induces charring effects. Therein, the condensed mechanism plays a critical role, leading to an increase in char yield from 4.36 % up to 12.94 %.

The formation of a compact char layer prevents the material from burning (See **Figure S41g-h**). Simultaneously, the fire growth rate (FGR) decreases as the fire performance index (FPI) increases with the incorporation of HBPPB. Moreover, LOI (limiting oxygen index) and UL-94 demonstrate improved fire-retardant performance (27.2% LOI and UL-94 V1 rating) for EP-9 compared to the thermoset EP (**Table S11**).

Table S11 LOI and UL-94 rating of EP and its blends with HBPPB

Samples	E51 (g)	MTHPA (g)	DMP-30 (g)	HBPPB (g)	LOI (vol %)	UL-94 Rating
EP	70.0	56.0	0.7	-	18.4	NR
EP-3	70.0	56.0	0.7	3.9	22.8	V1
EP-6	70.0	56.0	0.7	8.1	23.8	V1
EP-9	70.0	56.0	0.7	12.5	27.2	V1
EP-12	70.0	56.0	0.7	17.3	26.8	V1

The condensed fire-retardant action dominates due to the decrease in average effective heat of combustion (av-EHC). SEM, XPS and Raman spectroscopy were employed to further assert it. Firstly, SEM images displays the microsurface of residue char. In the controlled EP, underlying material presents a typical micro-porous feature with crack and fragile external char covering the surface, facilitating the continuous supply of heat and fuels, sustaining the fire until burning out. Conversely, at the same SEM scale, EP-9 (**Figure S41h**) shows an intumescent and condensed char layer, with macropores and cracks eliminated on polymer surface. Therefore, HBPPB plays a positive role in condensed mechanism, insulating flame and heat transfer to protect the underlying material. Furthermore, the chemical constitutions of char were studied by XPS technique, determining the remained phosphorus in char residue (**Figure S41i**). The signal of P2p can be identified in high-resolution XPS, fitting two peaks at 133.9 eV (P-C/P-O) and 132.6 eV (-P=O). In the spectrum of O1s, three deconvoluted peaks at 533.5 eV, 532.7 eV and 531.9 eV correspond to C-OH, O-C/P and C=C/P, respectively. In terms of C1s, the peaks at 288.6 eV, 285.3 eV and 284.8 eV are assigned to C=O, C-O-C/C-O-P and C=C/C-C.[24] Such results indicate that phosphorus ester involved in the char-forming to generate a stable and compact char. Moreover, Raman spectra show a low intensity ratio of D and G bands ($I_D : I_G$) in EP-9, indicating the higher graphitization degree of its residue char. (**Figure S42**).

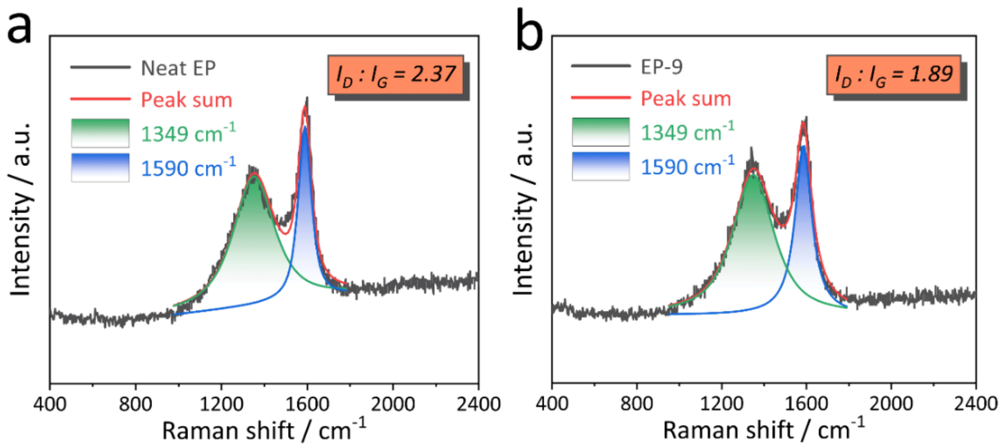

Figure S42 Raman spectra for the char surface of a) EP and b) EP-9 vitrimer.

S3. Supporting Movie

Movie S1 After heating in ethanol, EP-9 vitrimer sample shows transformative property in material nature from stiff vitrimer to elastomer that can be casually deformed and recovered as will at room-temperature.

References:

[1] A. P. Côté, A. I. Benin, N. W. Ockwig, M. O'Keeffe, A. J. Matzger, O. M. Yaghi, *Science* **2005**, 310, 1166.

[2] L. Guo, L. Yan, Y. He, W. Feng, Y. Zhao, B. Z. Tang, H. Yan, *Angew. Chem. Int. Edit.* **2022**, 61, e202204383.

[3] Y. Zhang, H. Yan, G. Feng, R. Liu, K. Yang, W. Feng, S. Zhang, C. He, *Compos. B-Eng.* **2021**, 222, 109043.

[4] T. Cai, L. Yuan, G. Liang, H. Wang, A. Gu, *Mater. Chem. Phys.* **2019**, 234, 67.

[5] Y. Zhang, R. Liu, R. Yu, K. Yang, L. Guo, H. Yan, *Compos. B-Eng.* **2022**, 242, 110101.

[6] J. Otera, *Chem. Rev.* **1993**, 93, 1449.

[7] Y. Yang, E. M. Terentjev, Y. Wei, Y. Ji, *Nat. Commun.* **2018**, 9, 1906.

[8] M. F. J. Mabesoone, A. R. A. Palmans, E. W. Meijer, *J. Am. Chem. Soc.* **2020**, 142, 19781.

[9] Z. Chen, Y. Chen, Y. Guo, Z. Yang, H. Li, H. Liu, *Adv. Funct. Mater.* **2022**, 32, 2201009.

[10] C. He, S. Shi, D. Wang, B. A. Helms, T. P. Russell, *J. Am. Chem. Soc.* **2019**, 141, 13753.

[11] S. S. Klara, P. O. Saboe, I. T. Sines, M. Babaei, P. Chiu, R. DeZorzi, K. Dayal, T. Walz, M. Kumar, M. S. Mauter, *J. Am. Chem. Soc.* **2016**, 138, 28.;

[12] X. Ouyang, Y. Yang, G. Zhu, X. Qiu, *Chinese Chem. Lett.* **2015**, 26, 980.

[13] L. Tang, J. Zhang, Y. Tang, Y. Zhou, Y. Lin, Z. Liu, J. Kong, T. Liu, J. Gu, *Compos. B-Eng.* **2020**, *186*, 107827.

[14] Y. Wang, K. Kou, G. Wu, L. Zhuo, J. Li, Y. Zhang, *Polymer* **2015**, 77, 354.

[15] M. Capelot, M. M. Unterlass, F. Tournilhac, L. Leibler, *ACS Macro. Lett.* **2012**, 1, 789.

[16] B. R. Elling, W. R. Dichtel, *ACS Cent. Sci.* **2020**, 6, 1488.

[17] J. Luo, Z. Demchuk, X. Zhao, T. Saito, M. Tian, A. P. Sokolov, P. Cao, *Matter* **2022**, 5, 1391.

[18] J. P. Brutman, P. A. Delgado, M. A. Hillmyer, *ACS Macro. Lett.* **2014**, 3, 607.

[19] M. Delahaye, J. M. Winne, F. E. Du Prez, *J. Am. Chem. Soc.* **2019**, 141, 15277.

[20] M. Capelot, M. M. Unterlass, F. Tournilhac, L. Leibler, *ACS Macro Lett.* **2012**, 1, 789.

[21] X. Liu, X. Zhao, W. An, R. Du, G. Wu, S. Xu, F. Zhang, Y. Wang, *Mater. Horiz.* **2022**, 14, 2993.

[22] S. Huo, P. Song, B. Yu, S. Ran, V. S. Chevali, L. Liu, Z. Fang, H. Wang, *Prog. Polym. Sci.* **2021**, 114, 101366.

[23] Z. Ma, J. Zhang, C. Maluk, Y. Yu, S. M. Seraji, B. Yu, H. Wang, P. Song, *Matter* **2022**, 5, 911.

[24] G. Ye, S. Huo, C. Wang, Q. Shi, L. Yu, Z. Liu, Z. Fang, H. Wang, *Compos. B-Eng.* **2021**, 227, 109395.
